# HiCanu: accurate assembly of segmental duplications, satellites, and allelic variants from high-fidelity long reads

**DOI:** 10.1101/gr.263566.120

**Published:** 2020-09

**Authors:** Sergey Nurk, Brian P. Walenz, Arang Rhie, Mitchell R. Vollger, Glennis A. Logsdon, Robert Grothe, Karen H. Miga, Evan E. Eichler, Adam M. Phillippy, Sergey Koren

**Affiliations:** 1 Genome Informatics Section, Computational and Statistical Genomics Branch, National Human Genome Research Institute, National Institutes of Health, Bethesda, Maryland 20894, USA;; 2 Department of Genome Sciences, University of Washington School of Medicine, Seattle, Washington 98195, USA;; 3 Pacific Biosciences, Menlo Park, California 94025, USA;; 4 UC Santa Cruz Genomics Institute, University of California, Santa Cruz, California 95064, USA;; 5 Howard Hughes Medical Institute, University of Washington, Seattle, Washington 98195, USA

## Abstract

Complete and accurate genome assemblies form the basis of most downstream genomic analyses and are of critical importance. Recent genome assembly projects have relied on a combination of noisy long-read sequencing and accurate short-read sequencing, with the former offering greater assembly continuity and the latter providing higher consensus accuracy. The recently introduced Pacific Biosciences (PacBio) HiFi sequencing technology bridges this divide by delivering long reads (>10 kbp) with high per-base accuracy (>99.9%). Here we present HiCanu, a modification of the Canu assembler designed to leverage the full potential of HiFi reads via homopolymer compression, overlap-based error correction, and aggressive false overlap filtering. We benchmark HiCanu with a focus on the recovery of haplotype diversity, major histocompatibility complex (MHC) variants, satellite DNAs, and segmental duplications. For diploid human genomes sequenced to 30× HiFi coverage, HiCanu achieved superior accuracy and allele recovery compared to the current state of the art. On the effectively haploid CHM13 human cell line, HiCanu achieved an NG50 contig size of 77 Mbp with a per-base consensus accuracy of 99.999% (QV50), surpassing recent assemblies of high-coverage, ultralong Oxford Nanopore Technologies (ONT) reads in terms of both accuracy and continuity. This HiCanu assembly correctly resolves 337 out of 341 validation BACs sampled from known segmental duplications and provides the first preliminary assemblies of nine complete human centromeric regions. Although gaps and errors still remain within the most challenging regions of the genome, these results represent a significant advance toward the complete assembly of human genomes.

Genome assembly is the process of reconstructing continuous genomic regions from shorter overlapping fragments, called reads (Miller et al. 2010; Nagarajan and Pop 2010). Recently, long-read sequencing technologies have significantly simplified assembly by generating multikilobase reads, which span most common genomic repeats (Chin et al. 2013; Koren et al. 2013; Koren and Phillippy 2014; Gordon et al. 2016; Bickhart et al. 2017; Kronenberg et al. 2018). Despite the per-base error rate of the input reads exceeding 10%, state-of-the-art assembly methods are able to resolve instances of longer repeats with sequence divergence as low as 2% (Koren et al. 2017; Kolmogorov et al. 2019). However, a significant fraction of the human genome is represented by long segmental duplications (SDs) of higher sequence identity. According to the current annotation of the human reference (Bailey et al. 2001, 2002), ∼208 Mbp of sequence is contained within repeats >20 kbp with sequence identity >98%. Low accuracy of the long-read technologies has also made continuous reconstruction of individual haplotypes very challenging because humans can average less than one heterozygous variant per 1 kbp. Typical assembly strategies collapse the genome first and phase afterward by calling variants, partitioning the reads, and reassembling (Chin et al. 2016; Seo et al. 2016). State-of-the-art methods integrate different sequencing technologies (Chaisson et al. 2019; Kronenberg et al. 2019) or parental information (Koren et al. 2018) to obtain chromosome-scale, haplotype-resolved assemblies. However, these approaches have the downside of collapsing multicopy repeats in the assembly or not resolving alleles that differ at only a few positions.

Recently, Pacific Biosciences (PacBio) introduced a new data type, referred to as HiFi reads (Wenger et al. 2019). The process of generating HiFi reads involves DNA fragmentation, adapter ligation and fragment circularization, and multipass sequencing of the circularized fragments. The resulting signal is then computationally processed to obtain an accurate consensus sequence for each individual fragment. To ensure that each fragment undergoes sufficient sequencing passes to obtain a high consensus accuracy, HiFi sequencing libraries are size selected for a target fragment size (currently up to 25 kbp).

Although the resulting read lengths are modest by the modern long-read sequencing standards—PacBio CLR reads frequently exceed 50 kbp, and ultralong Oxford Nanopore Technologies (ONT) reads can exceed even 100 kbp (Jain et al. 2018b), HiFi is a major leap forward in terms of long-read read accuracy. As the accuracy of other long-read technologies have not exceeded 95%, the median accuracy of current HiFi reads can exceed 99.9% (>Q30), making them a promising data type for separating highly similar repeat instances and alleles.

Early studies adopting HiFi sequencing showed improved variant calling and repeat resolution (Wenger et al. 2019; Vollger et al. 2020). However, these early assemblies were limited to resolving repeats with >1% sequence divergence, owing to limitations of existing tools (Wenger et al. 2019). The recently developed Peregrine assembler (Chin and Khalak 2019) greatly reduced assembly runtime and improved consensus accuracy, removing the need for postprocessing, but did not address the issue of suboptimal repeat resolution or allele separation. Other recent work combined HiFi sequencing with complementary data types, such as parental information (Wenger et al. 2019), Hi-C (Garg et al. 2019), and Strand-seq (Porubsky et al. 2019), to obtain chromosome-scale, haplotype-resolved assemblies.

In the following sections, we present HiCanu, a modification of the Canu assembler (Koren et al. 2017) designed to take full advantage of the high accuracy of HiFi reads. We evaluate HiCanu's ability to resolve near-identical genomic repeats, with a focus on centromeric repeats and SDs by comparing our results to other HiFi and recent ultralong Oxford Nanopore-based human assemblies (Miga et al. 2020; Shafin et al. 2020). Furthermore, we evaluate HiCanu's ability to capture both alleles in large phase blocks in a diploid human genome.

## Results

### HiCanu overview

**Figure 1.**
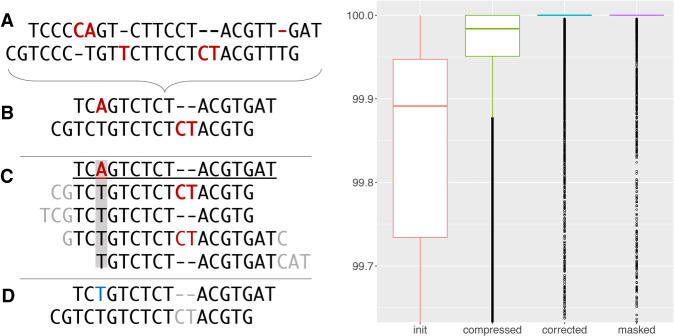
Impact of HiCanu processing on observed read quality. (*A*) Two hypothetical reads are shown with sequencing errors highlighted in red. (*B*) The first step of HiCanu is to compress homopolymers, which obscures homopolymer length errors but retains enough information to accurately distinguish reads from different genomic loci. (*C*) Overlaps are then computed for the compressed reads, and remaining errors are identified by examining the alignment pileups (gray rectangle). (*D*) Finally, after correcting the identified errors (blue) and ignoring indels in regions of known systematic error (gray), the resulting overlap is 100% identical. (*Right*) Sequence identity of reads from a 20-kbp HiFi library measured against the CHM13 Chromosome X reference sequence v0.7 (Miga et al. 2020) after each step of HiCanu processing (Supplemental Note 1). Separate boxplots are shown for initial raw HiFi reads (init), homopolymer-compressed reads (compressed), OEA-corrected reads (corrected), and corrected reads after ignoring differences in microsatellite repeats (masked). The median read identity, indicated by solid segments, increases from <99.9% to 100% (note the plot shows *y*-range of 99.65%–100%). Supplemental Table S1 also shows how HiCanu processing increases the percentage of perfectly aligned (100% identity) HiFi reads from <1% to >97%.

HiCanu builds on the original Canu pipeline, replacing or significantly modifying its core methods. Here we provide an overview of the new pipeline, highlighting the introduced changes, although a more detailed description of individual steps can be found in the Methods section. Whereas the original Canu pipeline starts with read self-correction, which can homogenize reads from different alleles or near-identical repeat instances, HiCanu begins by compressing all consecutive copies of the same nucleotide to a single base (e.g., “AA…” becomes “A”). In accordance with the earlier observation that misestimation of homopolymer length is the primary error mode of HiFi technology (Wenger et al. 2019), the resulting homopolymer-compressed reads (or “compressed reads” for short) accurately encode the transitions between different bases of the underlying genomic regions. The compressed reads are then trimmed based on their overlaps to other reads to remove any chimeric sequences or sequencing adapters (see overlap based trimming in Koren et al. 2017), and the overlaps are recomputed on the trimmed reads. The overlap error adjustment (OEA) module (Holt et al. 2002; Koren et al. 2017) examines read overlap pileups to identify remaining sequencing errors in the individual reads and recomputes overlap alignment identities. Following our observation that microsatellite repeat arrays are also prone to HiFi read errors, the OEA procedure was modified to ignore any differences within these regions when computing the final alignment identity of two overlapping reads. Compared with the initial reads (Fig. 1A), homopolymer compression (Fig. 1B), pileup-based read correction (Fig. 1C), and ignoring differences in microsatellite repeats (Fig. 1D) result in a drastic reduction of observed sequencing errors. Draft contigs are then formed from the adjusted overlaps using Canu's Bogart module (Koren et al. 2017), modified to better handle heterozygous variants and consider only high-identity read overlaps. The final contig sequences are obtained by computing a consensus across the original, uncompressed reads, arranged according to the layout of their compressed versions. Similar to many modern assemblers, when faced with a diploid genome, HiCanu outputs contigs as “pseudohaplotypes” that preserve local allelic phasing but may switch between haplotypes across longer distances. A single set of contigs representing all resolved alleles is output regardless of ploidy, and additional processing with a tool such as Purge_dups (Guan et al. 2020) is required to partition the contigs into primary and alternate allele sets.

### *Drosophila* genome assembly

We first evaluated HiCanu on a 24-kbp HiFi library from a *Drosophila melanogaster* F1 hybrid (ISO1×A4; see Data access). To match typical coverage, the HiFi data set was down-sampled to 40× and assembled with the HiFi-specific tools, HiCanu and Peregrine (Chin and Khalak 2019), as well as the conventional long-read assembler Canu. Canu was chosen as it was previously shown to achieve the highest assembly continuity and superior repeat resolution among other popular long-read assemblers on HiFi data (Wenger et al. 2019). For comparison, we also include a Canu assembly of 200× PacBio single-pass reads (CLR) for the same organism. Contigs <50 kbp were filtered from the assemblies in order to exclude low-quality sequences consisting of only a few reads.

Total assembly size varied between HiCanu (301 Mbp), Canu (293 Mbp), Peregrine (162 Mbp), and CLR (294 Mbp). Besides Peregrine, the assembly sizes were more than twice that of the 144-Mbp *D. melanogaster* haploid reference genome (Hoskins et al. 2015), suggesting that both haplotypes of the highly heterozygous F1 were successfully assembled (heterozygosity estimated at 0.7% by GenomeScope) (Supplemental Fig. S1; Vurture et al. 2017). The large fraction of duplicated BUSCO (Waterhouse et al. 2018) genes also supported the hypothesis that the assemblies captured alleles from both haplotypes (Supplemental Table S2). To facilitate like-for-like comparison of all assemblies, we ran Purge_dups (Guan et al. 2020) to identify alleles and divide the assemblies into primary and alternate contig sets (Methods). Assembly statistics were then computed for both contig sets and the results summarized in Table 1. Per-base consensus accuracy was estimated using Merqury (Rhie et al. 2020) with Illumina sequencing data from the *D. melanogaster* F1 parental strains (Supplemental Table S2; Supplemental Note 2).

**Table 1.**
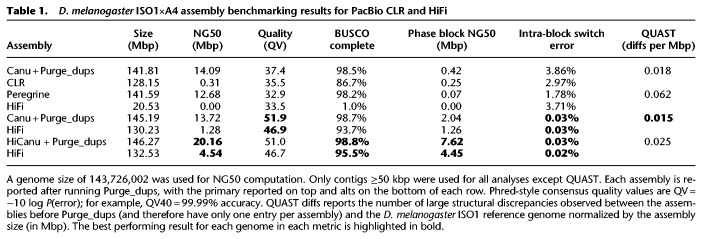
*D. melanogaster* ISO1×A4 assembly benchmarking results for PacBio CLR and HiFi

The primary contig sets across all assemblies reported high BUSCO completeness (>98%). BUSCO duplication values were <2% across all contig sets. The HiCanu primary contig set was noticeably more continuous than any other assembly as measured by NG50 (N such that half the haploid genome size is represented by contigs of this size or greater). Canu and HiCanu showed very similar per-base consensus accuracy, radically improving on both Peregrine and CLR assemblies. The Peregrine assembly collapsed both haplotypes together and output few alternate contigs (total length <21 Mbp). HiCanu improved over all other assemblies with respect to the total size, BUSCO completeness, and continuity of the alternate set (including a threefold improvement in NG50 over Canu).

To assess the integrity of the assemblies, we used QUAST v5.02 (Gurevich et al. 2013) to compare the assemblies against the chromosome arms of the *D. melanogaster* ISO1 reference. Because Purge_dups can split and/or trim the initial contigs but has a negligible effect on continuity, we report structural correctness of the original assemblies. Considering that one of the haplotypes (derived from the A4 parental strain) is expected to differ significantly from the reference, we adjusted QUAST's parameters to detect only large-scale genomic differences (Methods). Although the HiCanu assembly reported three more structural discrepancies than Canu (seven vs. four), it maintained the highest NG50 and alternate contig BUSCO completeness.

HiFi reads alone cannot be used to infer phasing across homozygous regions longer than the read length, so the contigs produced by HiCanu (and Canu) represent “pseudohaplotypes,” which may switch between haplotypes. However, for highly heterozygous genomes with short regions of homozygosity, HiCanu is expected to produce a low number of haplotype switches and mostly preserve long-range phasing. We used Merqury (Rhie et al. 2020) to split the initial contigs into continuous phase blocks, based on haplotype-specific *k*-mer markers inferred from parental Illumina reads (Supplemental Note 2). As a baseline, we considered a haplotype-resolved assembly produced by TrioCanu (Koren et al. 2018) generated using a combination of CLR reads and parental Illumina data. The HiCanu primary (alternate) contig set has an estimated phase block NG50 of 7.62 Mbp (4.45 Mbp), a maximal block length of 25.4 Mbp (10.1 Mbp), and a low percentage of discordant markers within predicted haplotype blocks (switch rate) of 0.03% (0.02%). For comparison, the TrioCanu assembly has a paternal-ISO (maternal-A4) phase block NG50 of 13.9 Mbp (21.39 Mbp), a max block size of 24.7 Mbp (27.7 Mbp), and an intra-block switch rate of 0.1% (0.04%). In contrast, the phase blocks of all other considered assemblies are much less continuous (at least a 3.5-fold drop in phase block NG50 compared to HiCanu) and, in the case of Peregrine and CLR assemblies, a much higher switch error (Table 1).

### Human genome assemblies

We next ran HiCanu, Canu, and Peregrine on three different human data sets (see Data access): a 20-kbp library of the completely homozygous cell line CHM13 (Kronenberg et al. 2018; Miga et al. 2020; Vollger et al. 2020), a 15-kbp library of the Ashkenazic cell line HG002 from the Personal Genome Project (Church 2005; Wenger et al. 2019), and a combined library (12% 10 kbp, 62% 15 kbp, 26% 20 kbp) for the Puerto Rican cell line HG00733 from the 1000 Genomes Project (The 1000 Genomes Project Consortium 2012; Porubsky et al. 2019). All data sets consist of approximately 30× HiFi sequencing coverage. For the HG002 data set, we reused the best assembly from a recent study (Wenger et al. 2019) as it reflects a Canu 1.7.1 assembly before HiCanu's development and the associated improvements to Canu's core modules. We additionally included the most continuous published (pseudohaplotype) assemblies of the same genomes, which relied on ultralong Oxford Nanopore reads to achieve state-of-the-art repeat resolution (Miga et al. 2020; Shafin et al. 2020). As before, contigs <50 kbp were excluded from analysis. As the sizes of HiCanu assemblies for the diploid data sets HG002 and HG00733 were 5.30 Gbp and 5.46 Gbp, respectively, compared with a haploid genome size of 3.1 Gbp, we again postprocessed all diploid assemblies with Purge_dups and computed statistics for both primary and alternate contig sets.

Per-base consensus quality was again estimated by Merqury (Rhie et al. 2020) using Illumina data from the corresponding genome (Supplemental Note 2; Supplemental Table S3). To assess the structural correctness of the assemblies, we followed the methodology of Shafin et al. (2020). Namely, structural differences reported by QUAST v5.0.2 versus the human reference genome GRCh38 (Schneider et al. 2017) were postprocessed to ignore breakpoints in centromeric regions and annotated SDs, in order to reduce the number of false positives (Methods; Supplemental Table S3). As before, because Purge_dups may introduce or correct misassemblies as it modifies the contigs, the structural correctness assessment was performed on the original assemblies.

Primary contig summary statistics for the three human genomes are presented in Table 2. The continuity of HiCanu assemblies, as measured by NG50, exceeded that of all other HiFi-based assemblies and even rivaled the continuity of Nanopore ultra-long-read assemblies. Reported rates of structural differences for HiCanu was on par with the other assemblies. For consensus accuracy, the HiCanu primary contig sets exceeded QV50 (99.999% accuracy) and alternate contigs sets exceeded QV40 (99.99% accuracy), whereas the unpolished Nanopore assemblies failed to exceed QV30 (99.9%). Although Nanopore assemblies currently require polishing with complementary technologies to maximize consensus accuracy, we discourage polishing HiCanu HiFi assemblies, because the available polishing pipelines may map reads back to the wrong repeat copies and actually introduce errors during polishing.

**Table 2.**
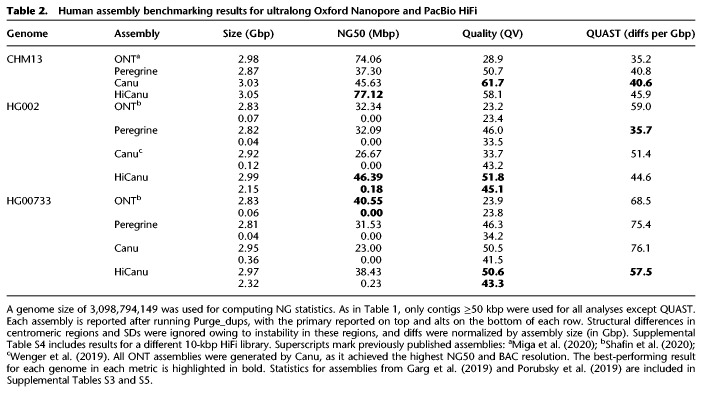
Human assembly benchmarking results for ultralong Oxford Nanopore and PacBio HiFi

The total length of the HiCanu alternate contig sets exceeded 2 Gbp, highlighting its ability to separate human alleles (corresponding values across other assemblies did not exceed 400 Mbp). The following section, “Human haplotype phasing,” further explores allele separation and phasing across these assemblies. The drastic improvements in consensus accuracy and allele separation for Canu versus HiCanu assemblies of HG002 is likely owing to Canu improvements and bug fixes made during the HiCanu development process, whereas the CHM13 and HG00733 assemblies represent the latest Canu version and the differences are less pronounced.

For CHM13 and HG00733 genomes, we additionally validated the assemblies against long continuous fragments of the corresponding genome, earlier reconstructed via bacterial artificial chromosome (BAC) sequencing (see Data access; no BACs were available for HG002). Many of these so-called “challenge” BACs were deliberately selected from genomic regions that pose significant assembly challenges (i.e., regions with SDs), making them useful for assembly benchmarking (Chin and Khalak 2019; Miga et al. 2020; Shafin et al. 2020; Vollger et al. 2020). Table 3 summarizes how well the challenge BACs are captured by different assemblies. To recognize a BAC as “resolved” within the assembly, we required 99.5% of the BAC length to be aligned to a single contig by minimap2 (Methods; Li 2018). Assembly sequence accuracy was measured as the median alignment identity of resolved BACs. Note that HiCanu resolved the highest number of BACs across all considered assemblies and also achieved the highest BAC alignment quality (Table 3; Supplemental Tables S4, S5).

**Table 3.**
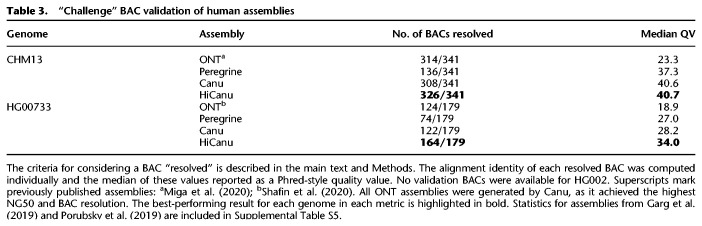
“Challenge” BAC validation of human assemblies

A deeper investigation of the unresolved CHM13 BAC sequences indicated that 11 BACs likely contain assembly errors or cloning artifacts themselves (Supplemental Note 3; Supplemental Figs. S2–S5). Manual inspection of HiFi read alignments did not reveal any standard misassembly signatures in the corresponding regions of the HiCanu assembly, providing evidence that HiCanu was correct in these cases and able to resolve 337 out of 341 (99%) of the CHM13 challenge BACs (Supplemental Table S6).

Although the challenge BACs are useful for validation, they do not represent the full landscape of human repeats. To further assess the ability of HiFi reads and different assemblers to resolve genomic repeats, we used the method of Vollger et al. (2019) to identify collapsed repeat instances in the CHM13 assemblies. We identified ∼21.7 Mbp of collapsed repeats corresponding to at least 56 Mbp of unresolved repetitive sequence. The HiCanu assembly had the lowest number of bases in regions identified as collapsed repeats, as well as the smallest amount of repetitive sequence predicted to be missing from the assembly (Supplemental Table S7; Supplemental Fig. S6). A complementary mapping-based analysis confirmed the comparatively high completeness of the HiCanu assembly and classified the majority (80%) of missing sequence as satellite repeats, suggesting good recovery of all other human repeat classes (Supplemental Fig. S6).

### Human haplotype phasing

When assembling a diploid genome, an assembler must choose to either collapse alleles into a single sequence or preserve them as two separate sequences. Collapsing heterozygosity results in a mosaic consensus that may not faithfully represent any allele and can introduce frameshifting errors within coding sequence.

HiCanu assemblies of the diploid human genomes included >2 Gbp of alternate contigs, with high BUSCO completeness for both primary and alternate contig sets (>94% and >75%, respectively) (Supplemental Table S8). We again used Merqury (Rhie et al. 2020) to analyze the phase blocks using parental Illumina data (Supplemental Note 2). The phase block NG50s of HiCanu primary (0.6 Mbp) and alternate (0.1 Mbp) contig sets were the highest across all considered assemblies (2.5-fold higher than next best) (Supplemental Table S8). Note that the human phase block NG50s are significantly shorter than for the *D. melanogaster* F1 hybrid but are longer than a typical human gene. For comparison, Supplemental Table S8 also includes statistics for the recently obtained haplotype-resolved assemblies of HG002 (Garg et al. 2019) and HG00733 (Porubsky et al. 2019). These recent studies have shown that multimegabase NG50 phase blocks can be obtained by integrating HiFi data with long-range linking information derived from Hi-C (Garg et al. 2019) or Strand-seq data (Porubsky et al. 2019).

To assess the phasing accuracy, we used a gold-standard variant set from the Genome in a Bottle (GIAB) consortium (Supplemental Table S9; Zook et al. 2019). HiCanu has an F_1_ score of 95.37%, which is >50% higher than the next best HiFi assembly and similar to another assembly combining both HiFi and Hi-C data (97.7%) (Garg et al. 2019). Finally, we validated the recovery of complex, clinically relevant alleles; we compared assembly typing results for the six classical human leukocyte antigen (HLA) genes (Dilthey et al. 2019) to the known alleles for HG002 and HG00733, obtained by previous studies (Supplemental Table S10; Chin et al. 2019; Shafin et al. 2020). Only HiCanu and TrioCanu were able to recover all alleles with 100% sequence identity (Supplemental Tables S10, S11). The HiCanu contigs expectedly switch between the haplotypes, but there is only one switch in the MHC region. The Hi-C-phased HG002 assembly from Garg et al. (2019) is phased across the length of the MHC region but contains consensus errors (e.g., both HLA-DRB1 alleles). The Strand-seq–phased HG00733 assembly from Porubsky et al. (2019) is also phased across the length of the MHC region but incorrectly represents HLA-A and HLA-B as homozygous (with both alleles in the assembly matching one ground-truth allele). Both the Garg et al. (2019) and Porubsky et al. (2019) methods rely on initially collapsed assemblies that are then phased using the long-range data. These results suggest that separation of haplotypes early in the assembly process (rather than trying to recover them from collapsed assemblies) may improve the accurate recovery of heterozygous variation.

### Complex regions of the CHM13 human genome

**Figure 2.**
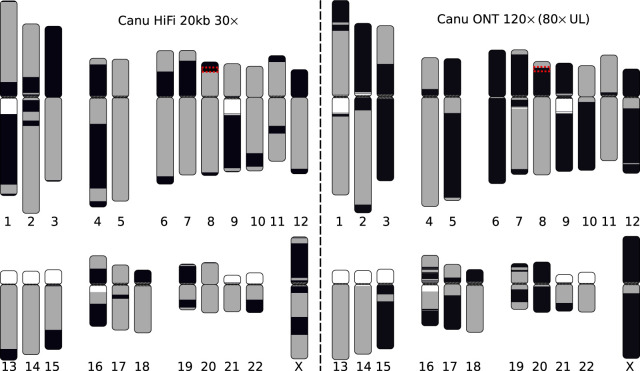
Visual representation of the most continuous HiFi-based and Nanopore-based assemblies of the CHM13 genome. HiCanu assembly of the 20-kbp HiFi data set (*left*) and Canu assembly of an ultralong Nanopore data set (*right*). White regions indicate gaps in the current reference genome, and each gray and black block indicates a continuous contig alignment. Color switches from gray to black represent either the end of a contig or an alignment break. Assemblies were aligned to GRCh38 using MashMap (Jain et al. 2018a), and plots were generated using coloredChromosomes (Böhringer et al. 2002) as previously described (Berlin et al. 2015; Jain et al. 2018b). Note that some chromosomes (e.g., Chr X) are better resolved by the Nanopore assembly owing to the presence of near-perfect repeats. At the same time, chromosomes containing more diverged repeats (e.g., Chr 7 and Chr 16) are better resolved by the HiFi assembly. We note that some gaps in the HiFi assembly are caused by sequence-specific biases of current HiFi sequencing protocols (Supplemental Note 4). The red box highlights the defensin beta gene family on Chromosome 8p23.1 which is split in both assemblies and detailed in Figure 4.

**Figure 3.**
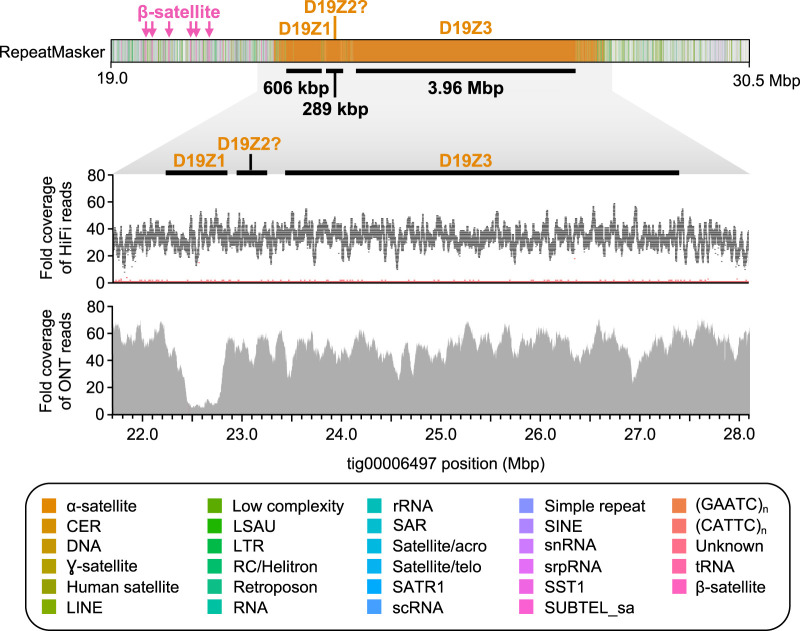
HiCanu assembly of the CHM13 Chromosome 19 centromere. RepeatMasker (Smit et al. 2013) of tig00006497 reveals three α-satellite HOR arrays that reside within the Chromosome 19 centromere (D19Z1, D19Z2?, and D19Z3; marked with black bars). These HOR arrays are 606 kbp, 289 kbp, and 3.96 Mbp in length, respectively, and are composed of a 13-mer, a complex higher-order HOR, and a dimeric HOR unit, respectively. The HOR repeat underlying D19Z2 shares limited sequence identity with the pG-A16 repeat previously described (Hulsebos et al. 1988; Choo et al. 1991; Finelli et al. 1996) and, therefore, is designated with a question mark. The α-satellite HOR arrays have relatively uniform coverage of HiFi and ultralong Oxford Nanopore data, except for a drop in Oxford Nanopore sequencing coverage over the D19Z1 array, which may be owing to a misassembly, read mismapping, or biases in sequencing. The HiFi coverage plot shows fold coverage of the most common base (black) and the second most common base (red).

The CHM13 HiCanu assembly (Tables 2, 3; Fig. 2) exceeded the predictions of our prior model of human assembly continuity (Supplemental Note 5; Supplemental Fig. S7). To validate this result, we focused on the performance of HiCanu within some of the most difficult-to-assemble regions of the genome, namely, centromeres and SDs. Unlike past assemblies of the human genome, including clone-based assemblies, HiCanu generated several contigs spanning mega-bases of satellite DNA. The CHM13 HiCanu assembly contains nine of 23 (39%) expected centromere regions: Chromosomes 2, 3, 7, 8, 10, 12, 16, 19, and 20 (Supplemental Note 6; Supplemental Table S11). The structure of these regions was consistent with an expectation of one or more higher-order repeat (HOR) array(s) flanked by more divergent tracts of monomeric satellite DNA (Willard and Waye 1987; Schueler et al. 2001; She et al. 2004). Mapped read depth of both HiFi and ultralong Oxford Nanopore data (Miga et al. 2020) across these contigs shows relatively uniform sequence coverage that spans the α-satellite HOR array(s) into the unique sequences on the p- and q-arms (Fig. 3; Supplemental Note 6; Supplemental Fig. S8). The structure and length of the centromeric HOR array(s) in each contig is highly concordant with those reported in the literature (for review, see McNulty and Sullivan 2018).

It is noteworthy that HiCanu generated a draft assembly of the CHM13 Chromosome 19 centromere (Fig. 3). Historically, this region has been considered to be one of the more challenging centromeres to reconstruct because it carries multiple HOR tracts and shares α-satellite sequences with the centromere regions from Chromosomes 1 and 5 (Hulsebos et al. 1988; Baldini et al. 1989; Pironon et al. 2010; Sullivan et al. 2017; McNulty and Sullivan 2018). HiCanu was not only able to assemble a contig that completely spans this centromere but also accurately distinguished three distinct HOR tracts (D19Z1, D19Z2, and D19Z3) (Supplemental Note 6; Supplemental Fig. S9). This contig revealed a more complete representation of the HOR structure of the D19Z1 HOR unit (13-mer vs. 10-mer) (Supplemental Figs. S9A, S10; Hulsebos et al. 1988; Puechberty et al. 1999), a Chromosome 19–specific dimeric HOR (D19Z3) (Supplemental Figs. S9B, S10; Baldini et al. 1989; Finelli et al. 1996), and two complex HORs (expected to represent D19Z2) (Supplemental Note 6; Supplemental Fig. S10). Alignment of HiFi sequence data to the corresponding HiCanu contig did not reveal any coverage anomalies (e.g., large dips or spikes) that could indicate the presence of structural errors. However, marker-assisted alignment of ultralong Oxford Nanopore data (Miga et al. 2020), an orthogonal data set, showed a coverage drop coinciding with the D19Z1 array. This may indicate a misassembly, mismapping of the noisy sequencing data, or biases in sequencing coverage. Because of the lack of a validated truth set in such regions, this will require extensive wet-laboratory validation and is left for future work.

Beyond the obvious challenge of centromere assembly, SDs represent another significant impediment and have been estimated to account for 68% of misassemblies and contig breaks in recent long-read genome assemblies, irrespective of the platform or assembly algorithm (Porubsky et al. 2019). To estimate the effect of SDs on the continuity of HiCanu assemblies, we aligned contigs from the CHM13 genome assemblies to the human reference genome (GRCh38) and tested if the ends of contigs mapped within SDs. Compared with the Canu, Peregrine, or ONT assemblies, HiCanu had the fewest contig breaks within SDs (*n* = 95) and the smallest overall fraction of contig ends mapping to SDs (49%) (Table 4). Of these 95 regions, 59 (62%) correspond to the longest (>10 kbp) and most identical (>98%) copy-number polymorphic duplicated regions of the human genome (Supplemental Fig. S11). These results indicate that SDs are better resolved using HiCanu; however, SDs still contribute disproportionately to the overall number of assembly breaks.

**Table 4.**
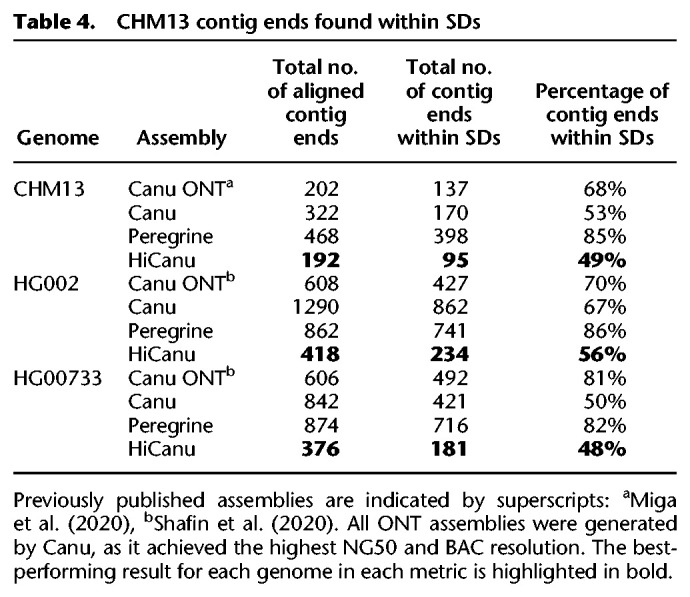
CHM13 contig ends found within SDs

**Figure 4.**
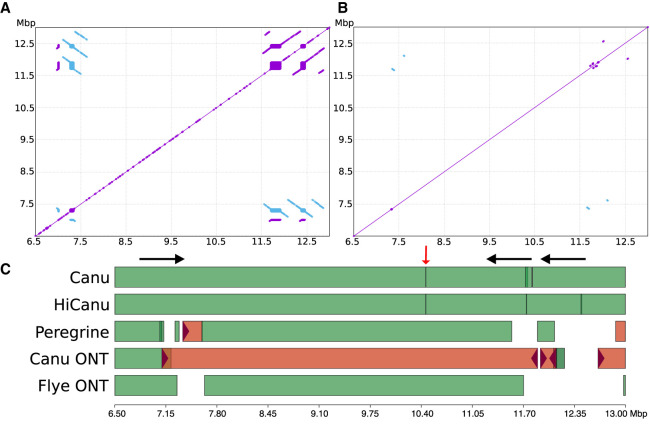
Chr 8 defensin beta cluster repeat structure and assembly comparison. (*Top*) NUCmer self-alignment dot plots (Kurtz et al. 2004) of the CHM13 reference defensin beta cluster at different alignment stringencies (Methods): (*A*) >7 kbp repeats at 98% identity. (*B*) >7 kbp repeats at 99.9% identity. Purple/blue indicates same/reverse strand matches. (*C*) Icarus (Mikheenko et al. 2016) visualization of contig alignments from both HiFi-based (Canu, HiCanu, Peregrine) and ultralong Nanopore-based assemblies (Canu ONT and Flye ONT) (Kolmogorov et al. 2019) produced by QUAST (Gurevich et al. 2013). White space in the alignment figure indicates the assembly was fragmented into short contigs (<50 kbp). Red color indicates misassembled contigs. The HiCanu assembly breaks at two of three SD instances that share high sequence similarity (black arrows) and at a region of systematic HiFi coverage depletion (red arrow).

The defensin beta gene family, a set of copy-number variable genes (e.g., *DEFB136*, *DEFB135*, etc.) mapping to two locations on Chromosome 8p23.1 (which we refer to as the defensin beta cluster), is a case in point. This ∼6-Mbp region plays an important role in immune function and disease (Weinberg et al. 2012; Mohajeri et al. 2016) and is known to be highly repetitive and difficult to assemble (Bakar et al. 2009). Previous reconstructions have relied on a BAC-by-BAC assembly approach (Mohajeri et al. 2016), and the first continuous assembly of this region in CHM13 was obtained via an independent approach using ultralong Nanopore data (GA Logsdon, MR Vollger, PH Hsieh, et al., in prep). Figure 4, A and B illustrates self-alignment dot plots at different stringencies of the defensin beta cluster from the T2T Chromosome 8 v3.0 assembly. Figure 4C shows the de novo assembled contig alignment and correctness against this draft. Both the Canu and HiCanu assemblies of the HiFi data consist of four contigs without structural errors. In contrast, the complex inverted repeat structures resulted in misassembled and fragmented contigs in all other evaluated assemblies.

The rightmost contig breaks in the HiFi assemblies are likely owing to the presence of long, nearly identical repeats, which would require either longer reads or a careful examination of repeat copy number to resolve. We also investigated the fragmentation of HiCanu and Canu contigs at position 10.4 Mbp, which is not part of any observed repeat structure. Alignment of the raw HiFi reads onto this region with minimap2 (Li 2018) revealed the presence of a 450-bp region covered by only two HiFi reads (Supplemental Fig. S12), with a coverage drop present in both the 10- and 20-kbp HiFi libraries. This coverage drop is flanked by a >250-bp simple-sequence repeat (AAAGG). Suspecting a possible bias in the HiFi datatype, we further examined Chromosome X, for which we have a complete CHM13 reference sequence available (Miga et al. 2020). On this chromosome, we identified at least four additional cases of HiFi coverage dropout, with all four instances associated with long, low-complexity (A/G or T/C-rich) sequences. As our HiFi assembly of Chromosome X is split into just 13 large contigs (Supplemental Fig. S13), this coverage bias appears to be a current weakness of the HiFi chemistry.

## Discussion

We have shown that HiCanu is capable of generating the most accurate and complete human genome assemblies to date and is able to achieve the resolution of repeats that are up to 99.99% identical. As a result, HiCanu surpasses prior HiFi and Nanopore ultra-long-read assemblies in terms of both repeat resolution and per-base consensus accuracy. HiFi data excels in resolving large highly-similar (but nonidentical) repeat instances. The remaining unresolved sequences seem to primarily represent satellite repeats (Supplemental Fig. S6). In particular, Figure 2 illustrates that HiCanu's reconstruction of human Chromosomes 1, 7, 9, and 16 notably improves continuity over the previous assembly of ultralong Nanopore reads (Koren et al. 2017; Kolmogorov et al. 2019; Shafin et al. 2020). These chromosomes contain several SDs exceeding 200 kbp in length, requiring high-fidelity reads to identify variants and separate the individual copies. HiFi data further enabled draft assemblies of nine centromeric regions, which are one of the final challenges of automated telomere-to-telomere assembly. Assembly of other centromeric regions is likely limited by a low frequency of unique markers compared with current HiFi read lengths. In contrast, Chromosome X has the highest count of large (>20 kbp) near-identical (>99.9%) repeats (Bailey et al. 2002) that were better resolved by long, spanning Nanopore reads. Thus, the two technologies are complementary at present, and the best technology depends on the specific characteristics of the repeats and haplotypes being assembled.

HiCanu's approach to read correction permits considering only the highest identity overlaps during contig construction and is general enough to be applied to other applications such as metagenomic assembly. Although HiCanu is not as fast as some of the competing methods, we note that the number of CPU hours required for assembly of a human genome is under 4000, which could be completed on any modern cloud platform in less than a day for a few hundred dollars. This is 30-fold less than recent Oxford Nanopore assemblies that required more than 100,000 CPU hours (Jain et al. 2018b; Shafin et al. 2020). At the time of writing, the most computationally expensive step of HiFi analysis is generating the data itself, because each individual HiFi read represents a consensus of multiple, aligned sequences of the same DNA molecule. Coupled with the instrument runtime and sequencing cost, HiCanu is a small fraction of the total project cost and duration (Supplemental Note 7). Most prior long-read assemblers have also required a final “polishing” step to improve consensus accuracy, which requires additional computation but can also introduce errors in repeat instances owing to ambiguous read mappings (Miga et al. 2020). Because of the initial accuracy of HiFi reads and because of the precise resolution of allelic variants and repeats, HiCanu assemblies do not require polishing.

When choosing HiFi, the library size should also be considered when beginning a sequencing project. Because HiFi read accuracy depends on the size of the sequenced fragments (shorter equals more passes and higher accuracy), one should consider the relative importance of read length versus accuracy. A metagenomic project may aim for shorter, higher accuracy reads to confidently identify low-abundance strains, whereas a vertebrate genome project may benefit from longer reads to span midsized identical repeats. We also identified an apparent bias in the current HiFi chemistry at low-complexity A/G (T/C) repeats, leading to coverage drops and assembly fragmentation. This issue warrants further investigation and may limit the applicability of HiFi sequencing to genomes with large stretches of such repeats. Thus, identifying optimal sequencing strategies and developing methods that can combine multiple technologies remains an area for future research.

HiCanu's diploid assemblies accurately capture both alleles in long haplotype blocks of very high quality (QV50). In particular, HiCanu consistently recovered both haplotypes for the six canonical MHC typing genes in the human genome, improving upon recently developed HiFi-based methods for haplotype-resolved assembly (Garg et al. 2019; Porubsky et al. 2019). However, because HiFi data alone do not provide long-range linking evidence, HiCanu's contigs represent pseudohaplotypes that typically require additional information and processing to achieve chromosome-scale phasing. Canu also does not assign contigs to haplotypes and requires postprocessing with a tool such as Purge_dups (Guan et al. 2020) to split the diploid assembly into primary and alternate alleles. Although recent studies have successfully integrated HiFi data with additional long-range linkage information (Garg et al. 2019; Porubsky et al. 2019), we do not expect that significant improvements in phasing can be achieved by HiFi-only assemblies without an increase of HiFi read lengths. One option is postprocessing of HiCanu assemblies by a haplotype-aware scaffolder, such as FALCON-Phase (Kronenberg et al. 2019), which could potentially correct haplotype switch events and deliver further improvements to phasing accuracy and assembly continuity. In general, we feel that HiFi contigs combined with Hi-C phasing and scaffolding is a promising recipe for phased telomere-to-telomere vertebrate genome assembly, and we plan to integrate these data types in future versions of Canu.

## Methods

### Mitigating errors in HiFi data

Although HiFi reads are highly accurate compared with other long-read sequencing technologies, they are not error free, which complicates the identification of reads originating from the same genomic loci during assembly. To identify and remove false read overlaps, we sought to increase the accuracy of HiFi data via read correction.

Wenger et al. (2019) observed that the majority of HiFi errors are in homopolymers, where the number of individually repeating nucleotides is incorrectly counted. To lessen the impact of such errors, HiCanu modifies the input reads by compressing every homopolymer to a single nucleotide. Our approach is similar to run-length encoding (RLE), which has been previously applied to 454 (Miller et al. 2008), PacBio CLR (Li 2016, 2018; Ruan and Li 2020), and Oxford Nanopore (Shafin et al. 2020) reads. However, HiCanu does not explicitly store the lengths of the compressed homopolymer stretches and instead reverts back to the uncompressed reads when needed.

Although the transition to homopolymer-compressed sequence space can reduce the specificity of the read alignment search, the corresponding reduction in the number of observed errors in the reads allow for a more restrictive alignment identity threshold (based on empirical analyses, we require a minimum overlap identity of 99%). Subsequent steps are performed on the homopolymer-compressed sequences, whereas the detailed correspondence between positions of original and compressed versions is generated on the fly when necessary. Compressed reads are first subjected to overlap-based trimming (Koren et al. 2017). Although this step only affected one human assembly during development (HG002 sequel II system with pre-2.0 early access chemistry 15-kbp library available from https://github.com/human-pangenomics/HG002_Data_Freeze_v1.0) and does not appear to be necessary for newer HiFi data sets, we chose to enable it by default on all assemblies in this paper for consistency. This improvement suggests that a significant fraction of reads was structurally incorrect in the poorly performing library owing to a low-quality sequencing library. Because other libraries did not show this problem, it is likely future versions of the HiCanu pipeline can skip this step and reduce runtime by >60%.

To further reduce the influence of the errors in compressed HiFi reads, we have updated the OEA module of Canu (Holt et al. 2002; Koren et al. 2017). This module identifies errors in individual reads by jointly considering all of their overlapping reads. Every such read votes for the nucleotides at the positions that it covers based on the pairwise alignment of the overlapping regions. A read's position is considered erroneous if no other reads support the original sequence and the majority of votes agree on a particular change (by default >50% and at least seven if there is a read supporting the original sequence). After the corrections are introduced, the alignment scores of the overlaps are recomputed, but the actual read sequences stored within the assembler are not modified as doing so would invalidate the previously computed overlap coordinates. Although our naive approach may not always be able to correct errors within highly-similar genomic repeats, such events are rare owing to the low number of errors in compressed HiFi reads and the high identity threshold used for gathering candidate overlaps.

Manual investigation of read alignments during HiCanu development revealed a previously unreported error mode in HiFi reads: incorrect repeat unit counts within microsatellite repeat arrays. Because the incorrect repeat counts are systematic and often supported by multiple reads, the conservative strategy described above is not able to correct them. Recognizing this, we modified the OEA procedure for recomputing overlap alignment scores to ignore sequence differences flanked by a microsatellite repeat in either read. Namely, the difference is ignored if five out of six nonoverlapping flanking *k*-mers are the same for any *k* ranging between two and six on either side (starting at zero to *k* − 1 bp from the difference). We note that this phenomenon deserves a deeper investigation, and our strategy can be improved to capture additional genomic differences, which are ignored by the current approach.

We evaluated the contribution of each of the above corrections using the recently completed CHM13 Chromosome X (Miga et al. 2020) as a reference. Raw, compressed, corrected, and masked 20-kbp HiFi reads were mapped and the mappings filtered to retain high-confidence alignments (Supplemental Note 1). Figure 1 shows the resulting alignment identity values, with each correction step boosting the identity of the aligned sequence. Each step (compression, correction, masking) contributes to this improvement (Supplemental Table S1; Supplemental Fig. S14). Although almost no (<1%) raw HiFi reads map error free, 97.23% of the compressed, corrected, and masked reads map without a single difference. Without correction (compression + masking only), reads have similar median error to just compression, and less than half have perfect alignment. As we did not control for reads mapping from other chromosomes and as the Chromosome X sequence itself is not error free, this likely represents a lower bound on the percentage of error-free reads. To extend beyond Chr X, we also estimated read accuracy using *k*-mers from short-read data for all human genomes and found correction improved read accuracy across all data sets (Supplemental Note 1).

### Bogart modifications

The Bogart module constructs a set of draft contigs from read overlap information. A detailed description is given by Koren et al. (2017). We describe here the modifications made for HiFi data.

#### Overlap identity threshold

Canu's initial overlap search uses a relaxed identity threshold to account for varying error rates between samples. Because overlap identities are changed by OEA and because we wished to avoid considering false-positive overlaps, Bogart first attempts to select a higher overlap identity threshold. Previously, Canu computed the identity of the best-scoring overlap on each side of every read (where score is defined as the number of matching bases in the overlap alignment) and set a threshold based on the median and MAD of the computed values (Koren et al. 2017). However, during the development, we realized that this way of computing the threshold was not informative for highly accurate reads because both the median and MAD were 100% across all tested data sets. Additionally, with the number of matching bases as a score, the read delivering the highest scoring overlap could come from a different haplotype in genomes with low heterozygosity. As a result, the selected threshold could inadvertently reflect the heterozygosity level of the organism rather than the accuracy of the reads. Based on empirical testing, we opted for an alternative two-step procedure. First, all overlaps with identity below the fixed value T (default 99.97% or three differences in 10,000 bp) are dropped. This step is aimed at removing from consideration the majority of the cross-haplotype overlaps even for low-heterozygosity organisms, for example, human heterozygosity rate of 0.1% (The 1000 Genomes Project Consortium 2012). Next, the identities of the highest scoring overlaps are collected as before, and the final threshold is set as the 90th% percentile of this sample. It is possible that 99.97% is too stringent given higher error reads. We could detect this condition when the 90th percentile is too close to 99.97% and rerun the overlap filtering. However, on all data sets evaluated to date, the chosen identity threshold was 100%. To support the desired overlap filtering stringency, the Canu codebase had to be modified to increase the precision with which the overlap identity values are stored.

#### Handling heterozygous differences

Bogart uses the filtered overlaps to identify and eliminate the reads likely representing sequencing artifacts and then constructs the *best overlap graph* (Miller et al. 2008), using the same overlap scoring function as before. This graph consists of the best-scoring overlap off both the 5′ and 3′ ends of each read, and the nonbranching paths within this graph form the preliminary layouts (arrangements of reads) that we refer to as *greedy contigs*. Bogart then inspects each greedy contig for long repeat instances that could have been incorrectly traversed. Repeats are detected by considering overlaps between the reads within and outside of the contig. If a suspected repeat has no reads spanning it or if there is a similar-length alternate read overlap, it is broken at the repeat boundary to avoid potential assembly errors as the method of Koren et al. (2017) to form the final *draft contigs*.

HiCanu aims to reconstruct long pseudohaplotype contigs (Vinson et al. 2005; Chin et al. 2016)—potentially switching between paternal and maternal alleles—and capture the alternative regions as shorter contigs. Unfortunately, the original Bogart approach described above led to the classification of extended homozygous regions within greedy contigs as unspanned repeats, leading to fragmentation of the pseudohaplotypes (Supplemental Fig. S15). In Canu, this behavior had been affecting only genomes with >1% heterozygosity, because below this threshold most heterozygous differences were implicitly hidden by the relatively permissive threshold on overlap identity. With the high-accuracy HiFi data, and a correspondingly high overlap identity threshold, this overfragmentation became an issue even for human levels of heterozygosity.

In HiCanu, Bogart has an additional step to identify contigs representing alternative alleles within the set of greedy contigs, which we refer to as *bubble contigs*. As suggested by the name, the bubble contigs are related to the bubble subgraphs, typically considered by most assemblers. Candidate bubbles are found by identifying reads in each contig that have overlaps to some other, larger, contig. A read within a smaller contig can be placed in the larger contig if the overlaps between it and the reads in the larger contig are below a specified threshold of similar quality to the previously incorporated overlaps (0.1% by default). If the placements for both the first and last reads of a candidate contig are correctly oriented and placed at approximately the correct distance in the larger contig (75%–125% of the candidate contig size), the candidate contig is flagged as a bubble and its reads are excluded from later repeat detection. This avoids fragmentation of otherwise structurally correct pseudohaplotype contigs. Similar strategies have previously been used in short-read assembly (Pevzner et al. 2001; Zerbino and Birney 2008; Li et al. 2010; Gnerre et al. 2011), scaffolding metagenomes (Koren et al. 2011; Ghurye et al. 2019), and long-read assembly (Chin et al. 2016). Bubble contigs are also explicitly marked in the final output; however, because placements are not always found, especially for longer, more heterozygous alleles, we recommend using a postprocessing tool such as Purge_dups (Guan et al. 2020) to classify alternate alleles and remove any false duplications.

### Consensus calculation

A consensus sequence is computed for all contigs using the uncompressed reads (trimmed to their good regions identified in compressed space). Canu originally used the layout produced by Bogart to estimate the position of each read within the contig and align it only to that location. Because the read layouts are now in homopolymer-compressed space, this strategy is unable to locate the read in uncompressed space. Instead, we compute the correspondence of each position in the compressed read to the original. This is used to update the read positions within the contig and expand the layout to uncompressed space. A modified version of the PBdagcon algorithm (Chin et al. 2013), with improved support for long contig sequences, is used to compute the final consensus sequence.

Currently, HiCanu will exclude erroneous reads from large contigs, but these erroneous reads may form their own short, low-coverage contigs. This can slightly reduce average assembly accuracy for homozygous genomes versus a more permissive strategy like that in Canu. However, Canu's more permissive approach will incorrectly mix haplotypes and similar repeat copies. Further HiCanu consensus gains are possible with better handling of erroneous reads and a more sophisticated approach for predicting homopolymer run length, similar to MarginPolish (Shafin et al. 2020).

### Previously generated data and assemblies

When available, previously published assemblies were downloaded and used. This included Oxford Nanopore UL Canu assemblies presented by Shafin et al. (2020) for HG0002 (80× Guppy HAC 2.3.5) and HG00733 (50× Guppy HAC 2.3.5), Canu + Racon assembly presented by Vollger et al. (2020), HG002 Canu assembly of HiFi reads presented by Wenger et al. (2019), Oxford Nanopore Canu assembly for CHM13 (40× + 80× UL Guppy HAC 3.1.5) presented by Miga et al. (2020), HiFi + Hi-C assemblies for HG002 presented by Garg et al. (2019), and HiFi + Strand-seq assemblies for HG0733 presented by Porubsky et al. (2019). In the remaining cases, assemblies were run locally on the NIH Biowulf cluster.

The *D. melanogaster* HiFi data are available from NCBI BioProject database (https://www.ncbi.nlm.nih.gov/bioproject/) at PRJNA573706 (SRR10238607; median: 24.4 kbp; mean: 24.4 kbp) and CLR (SRR9969843; median 13.3 kbp; mean: 17.2 kbp). Because of the high coverage, this data set was down-sampled to 40× HiFi data and 200× CLR data. These coverages represent ∼25% of the full run output. Because the exact parents of the F1 were not available, we used the previously generated short-read sequencing for binning and analysis (A4: SAMN00849823; ISO1: SRR6702604). The CHM13 Nanopore data are available at https:// s3.amazonaws.com/nanopore-human-wgs/chm13/nanopore/rel3/rel3.fastq.gz and Illumina at GitHub (https://github.com/nanopore-wgs-consortium/CHM13#10x-genomics-data). The HG002 Nanopore data are available at https://s3-us-west-2.amazonaws.com/human-pangenomics/index.html, HiFi at SRX5327410. HG002 and parent Illumina data are available from GIAB (Zook et al. 2016) at GitHub (https://github.com/genome-in-a-bottle/giab_data_indexes), we only used the 2×250 data sets. The HG00733 Nanopore data are available at https://s3-us-west-2.amazonaws.com/human-pangenomics/index.html, HiFi at ERX3831682. The Illumina data for HG00733 and parents were downloaded from the 1000 Genomes Project Consortium at https://www.internationalgenome.org/data-portal/sample (The 1000 Genomes Project Consortium 2012). The CHM13 Chromosome 8 reference assembly is available at GitHub (https://github.com/nanopore-wgs-consortium/CHM13#downloads).

### Software commands

HiCanu was run using Canu branch hicanu_rc with the following commands:

canu -assemble -p asm -d asm genomeSize=G -pacbio-hifi reads.fastq.gz

with G = 3.1 g for human and 150 m for *D. melanogaster*. This required 131 CPU hours and 16 GB of memory for *D. melanogaster*, 2780 CPU hours and 66 GB of memory for the CHM13 10-kbp library, 5000 CPU hours and 119 GB of memory for the CHM13 20-kbp library, 3999 CPU hours and 62 GB of memory for HG002, and 5233 CPU hours and 50 GB of memory for HG00733.

For the standard Canu assembles, Canu branch hicanu_rc ran with the following command:

canu -p asm -d asm genomeSize=G correctedErrorRate=0.015 batOptions=“-eg 0.01 -eM 0.01 -dg 6 -db 6 -dr 1 -ca 50 -cp 5” -pacbio-corrected reads.fastq.gz

with G = 3.1 g for human and 150 m for *D. melanogaster*. This required 232 CPU hours and 12 GB of memory for *D. melanogaster,* 3524 CPU hours and 80 GB of memory for the CHM13 20-kbp library, and 3836 CPU hours and 47 GB of memory for HG00733.

For CLR data Canu branch hicanu_rc was run with the following command:

canu -p asm -d asm genomeSize=150m corOutCoverage=100 batOptions=“-dg 6 -db 6 -dr 1 -ca 500 -cp 50” -pacbio-raw reads.fastq.gz

All HiFi assemblies required less than 12 wall-clock hours on the NIH Biowulf cluster quick partition with all jobs using <120 GB RAM. We reran HG002 on our cluster limiting the maximum concurrent CPUs to 288, which required 30 h. We estimated the cost of an AWS run using the c5d.18xlarge instance, which costs $3.456/h. Assuming four reserved nodes (for a total of 72 × 4 = 288 CPUs) and an average runtime of 4200 CPU hours with perfect parallelization, the run would complete in 14.5 h. We increase this by a factor of 2.0 to account for any nonparallelized steps based on the experiments above for a cost of $3.456 × 4 × 29 = $401. We note these estimates limited by differences in CPU and I/O between our cluster and AWS, as well as the overhead of waiting for a job to be scheduled on our cluster. The cost could also be reduced if additional nodes were spun up on-demand for the parallel portions of compute and spun down when not needed (as performed in Canu's DNAnexus implementation). We omit this from the estimate for simplicity. We also note that the assemblies could be completed faster if more nodes were allocated in parallel.

Peregrine assembler and SHIMMER ASMKit (0.1.5.3) was run with the command

yes yes | python3 /data/korens/devel/Peregrine/bin/pg_run.py asm \
     chm13.list 24 24 24 24 24 24 24 24 24 \
     ‐‐with-consensus ‐‐shimmer-r 3 ‐‐best_n_ovlp 8 \
     ‐‐output ./

This required 7 CPU hours and 29 GB of memory for *D. melanogaster*, 32 CPU hours and 347 GB of memory for the CHM13 10-kbp library, 58 CPU hours and 449 GB of memory for the CHM13 20-kbp library, 55 CPU hours and 407 GB for HG0002, and 63 CPU hours and 477 GB for HG00733.

#### Commands for defensin beta cluster and Chr X validation

HiCanu contigs flagged as bubbles were excluded from the analysis. MUMmer (Kurtz et al. 2004) 3.23 was used to identify repeats with the command:

nucmer ‐‐maxmatch ‐‐nosimplify
delta-filter -i 98 -l 10000

and high-stringency repeats

nucmer ‐‐maxmatch ‐‐noextend ‐‐nosimplify -l 500 -c 1000
delta-filter -i 99.9 -l 10000

QUAST alignments were generated as

quast.py -t 20 ‐‐large ‐‐skip-unaligned-mis-contigs ‐‐min-alignment 10000 ‐‐min-identity 98.0 ‐‐extensive-mis-size 5000 ‐‐min-contig 50000

Icarus was patched not to show breaks at “small indels” and “stretches of mismatches,” and used to visualize the resulting alignments.

#### Commands for RepeatMasker

RepeatMasker version 4.1.0 was run with the commands

RepeatMasker -pa 8 -q -species=mammal -xm -dir=asm.out asm.fasta

on each contig ≥50 kbp in the assembly. Centromeric arrays were identified by taking all hits marked as Satellite/centr and merging any hits within 100 bp of each other using BEDTools (Quinlan and Hall 2010). Resulting arrays >800 kbp were reported. There were nine internal arrays whose start and end coordinates were at least 500 kbp away from a contig end. These initial coordinates were manually curated based on reference alignments and are reported in Supplemental Table S12.

#### Commands for MHC typing

HLA*LA version commit 24930adadb0d2b6bcd69a271401dfc88a5d09f4d was run with the commands

HLA-ASM.pl ‐‐use_minimap2 1 ‐‐assembly_fasta $asm ‐‐sampleID $prefix ‐‐workingDir `pwd`/$prefix ‐‐truth reference_HLA_ASM/$truth

where $asm was the assembly, $prefix was a unique identifier, and $truth was either truth_HG002.txt or truth_HG00733.txt.

#### Commands for Purge_dups

Purge_dups version commit 8f580b41e6aa20c99383d6ff19b8689e93d7490e was run with the commands

python pd_config.py asm.fasta `pwd` <pb folder> <10x folder left blank> asm
minimap2 -I6G -xasm5 -DP asm.split asm.split > asm.split.self.paf
minimap2 -I6G -xmap-pb asm.fasta $line > pb.$jobid.paf (for each HiFi cell)
pbcstat pb.*.paf
calcuts PB.stat > cutoffs 2>calcults.log
purge_dups -2 -T cutoffs -c PB.base.cov asm.split.self.paf > dups.bed 2> purge_dups.log
get_seqs dups.bed asm.fasta > purged.fa 2> hap.fa

For *D. melanogaster*, an incorrect threshold was computed for the cutoffs owing to the entire genome being separated and so the cutoffs were manually adjusted to be

50 1 1 115 2 200.

The purged.fa output was then used as the primary set reported in the tables. To obtain the alternate set, we ran a second round of Purge_dups using hap.fa as the input assembly instead. This required an average of 20 CPU hours and 7 MB of memory for *D. melanogaster*, 59 CPU hours and 24 MB of memory for HG0002, and 74 CPU hours and 24 MB of memory for HG00733.

#### Commands for Merqury

Merqury version commit 154610d19ee6f4fead77da077af1ed7abdbe8866 was used. For each assembly and read set, canonical *k*-mers were built using meryl available as a binary within Canu:

meryl count k=<*k*-size> <reads.fastq.gz> output <genome>.*k*<*k*-size>.meryl
meryl count k=<*k*-size> <asm.fasta> output <asm>.*k*<*k*-size>.meryl

using *k* = 21 for humans and *k* = 18 for *D. melanogaster* based on (Fofanov et al. 2004). QV and *k*-mer completeness were obtained with

eval/spectra_cn.sh

which converts *k*-mer Jaccard to distance as previously described (Ondov et al. 2019) and to a Phred score (Ewing and Green 1998). Haplotype blocks were estimated by first building parent-specific *k*-mer databases. *K*-mers in each parental data set were counted as above, then subtracted to obtain parent-specific *k*-mers, and finally intersected with the child (in the case of human data sets in which child Illumina data was available) with

trio/hapmers.sh
trio/phased_block.sh

For further information see Supplemental Note 2 and https:// github.com/marbl/merqury/wiki.

#### Commands used for QUAST

QUAST 5.0.2 ran with the command

quast.py <asm> -o quast_results/<asm> -r <reference> -t 16 -s ‐‐large

Variants were filtered using the pipeline from (Shafin et al. 2020) to filter errors in varying sites, including known SVs (HG002 only available from GIAB) (Zook et al. 2020) at ftp://ftp-trace.ncbi.nlm.nih.gov/giab/ftp/data/AshkenazimTrio/analysis/NIST_SVs_Integration_v0.6/HG002_SVs_Tier1plusTier2_v0.6.1.bed):

python3 reference/quast_sv_extractor.py -q quast_results/<asm>/contigs_reports/all_alignments*tsv -c reference/centromere.bed -d reference/GRCh38_marked_regions.bed -s reference/empty

and

python3 reference/quast_sv_extractor.py -q quast_results/<asm>/contigs_reports/all_alignments*tsv -c reference/centromere.bed -d reference/GRCh38_marked_regions.bed -s reference/HG002_SVs_Tier1plusTier2_v0.6.1.bed

for HG002. We used https://www.ncbi.nlm.nih.gov/assembly/GCF_000001215.4 filtered to remove any unassigned sequences for *D. melanogaster* (Chr 2L, Chr 2R, Chr 3L, Chr 3R, Chr 4, Chr M, Chr X, Chr Y only) and https://hgdownload.soe.ucsc.edu/goldenPath/hg38/bigZips/hg38.fa.gz filtered to exclude alts and unaligned sequences (Chromosomes 1–22, X, Y, and MT only). Because no filtering file was available for *D. melanogaster,* we modified QUAST parameters to try to avoid false-positive misassembly counts with the command

quast.py <asm> -o quast_results/<asm> -r <reference> ‐‐large ‐‐min-alignment 20000 ‐‐extensive-mis-size 500000 ‐‐min-identity 90

#### Commands for BAC validation

We used the BAC validation pipeline available at GitHub (at https://github.com/skoren/bacValidation) run with default parameters. This pipeline aligns reads using minimap2 (Li 2018) and parses the SAM (Li et al. 2009) format to generate summary statistics. Output BAC identity was computed as the median across all BACs marked as correctly resolved. BAC libraries were downloaded from NCBI (CHM13: https://www.ncbi.nlm.nih.gov/nuccore/?term=VMRC59+and+complete, HG00733: https://www.ncbi.nlm.nih.gov/nuccore/?term=VMRC62+and+complete). HiFi read alignments to the assembly and BAC sequences were visualized with the Integrative Genomics Viewer (IGV) (Robinson et al. 2011).

#### Commands for variant analysis

We downloaded trio-phased GIAB (Zook et al. 2019) variant calls for HG002 from ftp://ftp-trace.ncbi.nlm.nih.gov/ReferenceSamples/giab/release/AshkenazimTrio/HG002_NA24385_son/NI STv3.3.2/GRCh38/HG002_GRCh38_GIAB_highconf_CG-Illfb-IllsentieonHC-Ion-10XsentieonHC-SOLIDgatkHC_CHROM1-22_v.3.3.2_highconf_triophased.vcf.gz. We ran dipcall followed by vcfeval to estimate SNP sensitivity and precision (Supplemental Table S8) with the commands

run-dipcall hg002_purge GRCh38_full_analysis_ set_plus_ decoy_hla.fa primary.fasta alts.fasta > hg002.mak
make -j1 -f hg002.mak
# exclude chrX/Y since there are no GIAB variants on them
gunzip -c hg002_purge.dip.vcf.gz |grep -v chrX |grep -v chrY |bgzip -c > hg002_purge.dip_ nohom.vcf.gz
# mark calls as homozygous alt in regions where only primary calls a variant and no alts map
gunzip -c hg002_purge.dip_nohom.vcf.gz | sed 's/GAP2/./;s/1|\./1|1/;s/ID=\./ID=GAP2/' | grep -v 'HET\|GAP\|DIP' | bgzip -c > hg002_purge.dip.vcf.gz
tabix hg002_purge.dip_nohom.vcf.gz
tabix hg002_purge.dip.vcf.gz
# measure statistics
rtg vcfeval -b HG002_GRCh38_GIAB_highconf_CG-Illfb-IllsentieonHC-Ion-10XsentieonHC-SOLIDgatkHC_CHROM1-22_v.3.3.2_highconf_triophased.vcf.gz -c hg002_purge.dip.vcf.gz -e HG002_GRCh38_GIAB_highconf_CG-Illfb-IllsentieonHC-Ion-10XsentieonHC-SOLIDgatkHC_CHROM1-22_v.3.3.2_highconf_noinconsistent.bed -t GRCh38_hs38d1.sdf -m annotate -o hom

To evaluate phasing, we evaluated the number of maternal and paternal variant calls out of the true positive calls in each contig and reported the total fraction of misphased variants (Supplemental Note 8).

#### Commands for identifying contig ends

Alignments were made between assemblies and GRCh38 using the following minimap2 command:

minimap2 ‐‐secondary=no -a ‐‐eqx -Y -x asm20 -s 200000 -z 10000,50 -r 50000 ‐‐end-bonus=100 -O 5,56 -E 4,1 -B 5

Contig ends that intersected SDs were identified by parsing the CIGAR string to find the location of contig ends and then by intersecting these locations with annotated SDs plus 10 kbp on either side from the UCSC Genome Browser using the following commands:

bedtools slop -i {segdups.bed} -b 10000 | bedtools merge -i - > {expanded.segdups.bed} && bedtools intersect -a {contig.ends.bed} -b {expanded.segdups.bed}

## Data access

All raw and processed sequencing data generated in this study have been submitted to the NCBI BioProject database (https://www.ncbi.nlm.nih.gov/bioproject/) under accession number PRJNA530776 (10 kbp: SRR9087597–SRR9087600; 20 kbp: SRR11292120–SRR11292123).

We have posted the down-sampled data sets, generated assemblies, and corrected CHM13 BAC sequences at https://obj.umiacs.umd.edu/marbl_publications/hicanu/index.html. HiCanu is implemented within the Canu assembly framework and is available as Supplemental Code and from GitHub (https://github.com/marbl/canu).

## Competing interest statement

R.G. is an employee and shareholder of Pacific Biosciences. E.E.E. is on the scientific advisory board of DNAnexus. All other authors have no competing interests to declare.

## Supplementary Material

Supplemental Material
