# Supplementary material for: HiCanu: accurate assembly of segmental duplications, satellites, and allelic variants from high-fidelity long reads
Source: Genome Res. 2020 Sep;30(9):1291–305. doi: 10.1101/gr.263566.120 (PMC7545148; doi:10.1101/gr.263566.120)
Supplement: Supplemental Material [file supp_30_9_1291__index.html]

HiCanu: accurate assembly of segmental duplications, satellites, and allelic variants from high-fidelity long reads — Supplemental Material 

# HiCanu: accurate assembly of segmental duplications, satellites, and allelic variants from high-fidelity long reads

## Supplemental Material

- Supplemental\_src.zip
- Supplementary\_Materials.docx
