# Supplementary material for: HiCanu: accurate assembly of segmental duplications, satellites, and allelic variants from high-fidelity long reads
Source: Genome Res. 2020 Sep;30(9):1291–305. doi: 10.1101/gr.263566.120 (PMC7545148; doi:10.1101/gr.263566.120)
Supplement: Supplemental Material [file supp_gr.263566.120_Supplemental_Material_.pdf]

Supplementary Materials for “HiCanu: accurate assembly of segmental duplications, satellites, and allelic variants from high-fidelity long reads”

Supplementary Materials for “HiCanu: accurate assembly of segmental duplications, satellites, and allelic variants from high-fidelity long reads” 1
- Supplementary Note 1: Evaluation of HiFi read accuracy 2
- Supplementary Note 2: Using *k*-mers for assembly evaluation and identifying haplotype-blocks within diploid assemblies 3
- Supplementary Note 3: CHM13 Challenge BAC Validation 4
- Supplementary Note 4: Identification of low-coverage gaps 5
- Supplementary Note 5: Human repeat modeling 6
- Supplementary Note 6: Centromere analysis and validation 7
- Supplementary Note 7: Estimate of AWS costs 8
- Supplementary Note 8: Variant phasing estimation 9
- Supplementary Figures 11
- Supplementary Tables 26

Supplementary Note 1: Evaluation of HiFi read accuracy

We took all 20 kbp HiFi sequences from the CHM13 cell line and aligned them to the v0.7 ChrX assembly. The reads were aligned with minimap2 (Li 2018) v2.17 with the command:

```
minimap2 -a -H -t 15 -x asm20
```

Reads were then homopolymer-compressed (and trimmed by Canu, though untrimmed reads showed a similar alignment identities) and aligned to the same reference. Finally, the compressed and trimmed reads were corrected by Canu's OEA module and aligned. Only primary unambiguous alignments were retained using the command:

```
samtools view -F 2304 -q 60
```

Then alignment identity values were computed by a custom script (https://github.com/snurk/bacValidation/blob/master/samToErrorRate.C), optionally ignoring the differences flanked by microsatellite repeat arrays. Only reads with a single alignment covering at least 99% of their length with identity >98% were evaluated. Note that identity may vary across the genome and these are summary statistics, as both sequencing errors and the correction procedure are region-specific.

To extend our analysis beyond CHM13 and ChrX, we also estimated read accuracy using *k*-mers. We build compressed *k*-mer databases for the Illumina data for each genome using meryl with the command:

```
meryl k=30 memory=40 threads=16 compress count $read_file output $read_file.meryl
meryl k=30 memory=40 threads=16 union-sum output G.k30.meryl *.meryl
meryl histogram G.k30.meryl > histogram
```

evaluated the histograms, and picked a minimum threshold for trusted *k*-mers (>10 for CHM13/HG002, >3 for HG0733) and counted *k*-mers in reads:

```
meryl threads=16 greater-than $threshold G.k30.meryl output G.k30_filtered.meryl
meryl-lookup -memory 40 -threads 16 -existence -sequence $reads -output G.kmers -mers
G.k30_filtered.meryl
```

and calculated identity as in Ondov et al. 2019 (see QV and *k*-mer completeness in Supplementary Note 2 below):

```
cat G.kmers |awk '{print $(NF-2)-$NF" "$(NF-2)}'| awk -v k=30 '{print (1-$1/$2)^(1/k)}'
```

The validation was limited by low coverage on HG0733, where the *k*-mer peak was at 12x (vs 30x for CHM13 and 60x for HG002). There are potential biases from both short-read coverage, which can mark true *k*-mers as incorrect, and identity estimates, which assumes a random error model. Lastly, the *k*-mer identity does not account for errors due to microsatellite repeat arrays. Thus, the measured identity is likely a lower bound. Despite these limitations, we found correction always boosted read identity and the fraction of perfectly covered reads. Compressed HiFi read median identity was 99.97% (99.98% CHM13, 99.98% HG002, 99.93% HG0733), with 8.7% perfectly covered (7.3% CHM13, 18.0% HG002, 1.8% HG0733). Post compression and correction it was 99.98% identity (99.99% CHM13, 99.99% HG002, 99.95% HG0733) and 14.7% covered (14.4% CHM13, 28.8% HG002, 2.9% HG0733).

Supplementary Note 2: Using *k*-mers for assembly evaluation and identifying haplotype-blocks within diploid assemblies

We used Merqury (Rhie et al 2020) to estimate assembly *k*-mer level QV, completeness, and phase block statistics. We computed an optimal *k*-mer size of 18 for the *D. melanogaster* and 21 for human, as in Fofanov et al., with a collision rate of 0.001 (automated in Merqury's `best_k.sh`). Since QV varies depending on the *k*-mer size used we also calculated QV for all genomes using a *k*-mer size of 31. The 31-mer QV was lower than 18-and 21-mer, by as many as 4.5QV points. However, the relative ordering of the assemblies remained stable.

QV and *k*-mer completeness

The similarity of an assembly to a read set is defined as the number of *k*-mers shared by *both* the assembly and the reads divided by the total number of *k*-mers found *only* in the assembly. This can be converted to a similarity as in Ondov et al. 2019:

$$i = (K_{shared} / K_{total})^{1/k}$$

which is akin to percent sequence identity and is converted to the Phred scale as:

$$QV = -10 \log_{10}(1-i)$$

Completeness is measured as the fraction of "reliable" *k*-mers in the read set present in an assembly. To determine reliable *k*-mers, we build the histogram of *k*-mer counts (multiplicity = number of times a *k*-mer is seen in the read set). The minimum reliable *k*-mer threshold is set to the lowest multiplicity with a positive slope in the histogram (automated in Merqury, with `build/filt.sh`)

Phase blocks

Haplotype-specific markers were determined using parental specific *k*-mers as previously done for trio binning (Koren et al. 2018). *K*-mer databases were built for each parental read and subtracted to obtain parent-specific markers. Reliable *k*-mers for each parent-specific database are selected as above. These databases were used for the *D. melanogaster*. For human datasets, the parent-specific *k*-mer databases were further intersected with the child's F1 Illumina data to only include parent-specific markers which were inherited by the child.

Phase blocks were obtained by querying each *k*-mer found in the assembly to each parental *k*-mer database. A phase block is defined as having at least 2 haplotype-specific markers from the same haplotype, allowing short-range "switches" to the other haplotype. We defined a short-range switch as at most 100 consecutive markers within a 20 kbp region. When more than 100 markers from the other haplotype are found or they span more than 20 kbp, a new block is created. The new block starts at the first inconsistent marker found. The switch error rate is defined as the fraction of markers from the wrong haplotype within all phase blocks.

Supplementary Note 3: CHM13 Challenge BAC Validation

We inspected all 15 of the CHM13 BACs which were not correctly resolved by the HiCanu assembly. We mapped all 20 kbp HiFi reads to the BACs and to the HiCanu assembly with minimap2:

```
minimap2 -t 32 -ax map-pb -r 2000 -m 3000 <reference.fasta> *.fastq.gz
```

We also checked the BACs for sequence similarity to the vector and *E. coli* sequences. We concluded that 11 of the sequences were incorrect or highly suspicious:

- AC278245.1 Low complexity sequence likely expanded or contracted within the BAC. (likely error)
- AC278709.1 BAC includes 2047 bp of cloning vector (error).
- AC278859.1 BAC includes 2047 bp of cloning vector (error).
- AC278792.1 Soft clipped 331bp (left) of sequence in the BAC that matches many other BACs in BLAST at perfect identity, untrimmed vector (error), and soft clipped (right) 4779 of cloning vector (error).
- AC278658.1 BAC includes 2047 bp of cloning vector (error).
- AC278258.1 BAC includes 872 bp of cloning vector (error).
- AC278368.1 BAC includes 2047 bp of cloning vector (error).
- AC279108.1 Insertion of 246 bases in the assembly supported by all HiFi data (likely error), Supplementary Figure 2
- AC278968.1 Lots of base mismatches and a large SV being called by all HiFi data. (likely error), Supplementary Figure 3
- AC278708.1 Soft clipped 331bp of sequence in the BAC that matches many other BACs in BLAST at perfect identity, likely untrimmed sequence of some kind. Clipped identically in GRCh38.p13 and HiFi and UL assemblies (likely error), Supplementary Figure 4
- AC278985.1 Lots of base mismatches and a large amount of soft clipping (2045). Clipped identically in GRCh38.p13 and HiFi and UL assemblies (likely error), Supplementary Figure 5

The adjusted CHM13 BAC resolution stats can be found in Supplementary Table 5.
We claim that only four BAC regions were unresolved by the HiCanu assembly:

- AC279099.1 Soft-clipped BAC extends past contig end
- AC279073.1 Lots of mismatches across the whole BAC and 7345 bp of soft clipped sequence extends past the contig end.
- AC270468.1 BAC extends past the contig end.
- AC278922.1 BAC split across two contigs

Supplementary Note 4: Identification of low-coverage gaps

All HiFi reads were mapped to the Chr8 and ChrX T2T assembly with minimap2 with the commands:

```
minimap2 -a -H -t 15 -x asm20
```

The HiCanu assembled contigs were also mapped to the T2T assembly with the same commands. We identified regions with <= 2-fold HiFi coverage coinciding with a break in the contig mapping. The regions were visually inspected in IGV to confirm simple-sequence repeats.

Supplementary Note 5: Human repeat modeling

We previously developed a model of human assembly continuity to predict the effects of read length and accuracy. Increased read length allows for more repeats to be spanned by reads that are uniquely anchored on either side, while increased read accuracy allows for more repeats to be separated based on sequence variants within the repeat. Using PacBio CLR or Nanopore data, existing algorithms have been able to separate repeats that are up to 98% identical at the sequence level by detecting variants within the repeats. Assuming a 20 kbp read length, this model predicts that improving repeat separation from 98% to 99.9% identical repeats would boost assembly NG50 by 1.8-fold (N such that 50% of the genome size is assembled in contigs of this size or greater). Thus, because of their improved ability to separate repeats, highly accurate 20 kbp reads are predicted to rival noisy 200 kbp reads in terms of assembly continuity (Supplementary Figure 7).

Supplementary Note 6: Centromere analysis and validation

HiFi data was aligned to the HiCanu assembly with pbmm2 v1.1.0 and the command:

```
pbmm2 align --preset SUBREAD -N 50 --min-length 3000 -r 50000 {input.ref} {input.reads} | samtools view -u -F 2308 - | samtools sort -o {output}
```

ONT rel3 data from (Miga et al. 2020) was aligned to the HiCanu assembly with minimap2 with the command:

```
minimap2 -ax map-ont -k15 -w5 -N 50 -r 10000 <asm.fasta> <reads.fastq>
```

and filtered unique markers as in (Miga et al. 2020). The resulting coverage was visualized with IGV and repeats within each array annotated using RepeatMasker.

The 606 kbp D19Z1 α-satellite array within the Chromosome 19 centromeric repeat is composed of a 2.25 kb (13-mer) HOR unit (Hulsebos et al. 1988), completing the repeat representation from the partial (10-mer) sequence available in Genbank (AJ295045.1). Further, we demonstrate that intra-array homogeneity is high, with units of the 13-mer HORs within the D19Z1 array 97.5% identical on average (Supplementary Figure 9a). The 3.96 Mbp D19Z3 α-satellite array is predicted to consist primarily of a dimeric HOR unit (Baldini et al. 1989). While the dimeric array on Chromosome 1, 5, and 19 centromeres (pC1.8 clone, Genbank M26919 and M26920) has demonstrated similar hybridization patterns (Baldini et al. 1989; Finelli et al.1996), these sequences shared only 93% sequence identity to the HiCanu D19Z3 HOR array, on average. This supports previous findings that chromosome-specific sequences are present in D19Z3 to distinguish these arrays at the sequence level (Pironon, et al. 2010). Here, we report a new dimeric α-satellite sequence for D19Z3 that is 95.7% identical, on average, between HOR units in the array. (Supplementary Figure 9b). Finally, the ~300 kbp D19Z2 array consists of two different HORs, but these repeat structures had not been characterized before. However, they share sequence similarity with the GJ211883.1 (cen5_2) and GJ211884.1 (cen5_4) alpha satellite reference models in GRCh38. Using the HiCanu assembly and the underlying raw HiFi data, we identified two complex D19Z2 HOR structures that are, themselves, comprised of α-satellite HORs. These complex HOR structures are organized as follows: 7-, 8-, 6-, 7-, 4-mer, and 6-, 6-, 3-, 4-, 4-, 7-, 4-, 4-, 2-mer (Supplementary Figure 10).

Supplementary Note 7: Estimate of AWS costs

Assuming a 30-hr movie (https://www.pacb.com/products-and-services/sequel-system/latest-system-release/), acquiring CHM13 data on four SMRTcells would require 120 hours. We obtained 3/6 NA12878 raw bam files for the subreads and converted them to HiFi using the CCS command v3.4.1:

```
        ccs --maxLength 21000 --minPasses 3 --numThreads 32 --polish --minPredictedAccuracy 0.99 input.bam
output.bam
```

The jobs took on average 7,136 CPU h and 13 GB memory. Thus, we estimated a total of 42,817 CPU h to convert all six cells to HiFi reads. This corresponds to 199 hours on a 36-core node per cell. Using a c5d.9xlarge (1.728/hr) instance to allow storing large BAM files locally, this would cost $2,055. Finally, we estimate the HiCanu runtime on AWS to be 29 hours versus 2 hours for Peregrine. The total runtime with HiCanu is 348 and with Peregrine is 321, a difference of 8%. For cost, we ignore reagent and consumable cost and focus only on computation. We estimated above the cost to generate HiFi reads on AWS as $2,055 and HiCanu as $400 vs $10 for Peregrine. The total cost with HiCanu is $2,455 and with Peregrine $2,065, a difference of 19%.

Supplementary Note 8: Variant phasing estimation

We used a custom script to check the number of maternal and paternal variants in each assembled contig:

```
# get all mappings for a contig
zcat ../hg002_purge.$HAP.paf.gz |grep -v decoy |grep -v chrUn |grep -v alt |grep $tigID |awk -v
MIN=0 -v MAX=0 '{if ($8 < MIN || MIN == 0) {MIN=$8; } if ($9  > MAX) {MAX=$9; } if (CHR != "" &&
CHR != $6) print "Error: cannot evaluate"; CHR=$6; } END {print CHR"\t"MIN"\t"MAX}' > tmp.bed

len=`zcat ../hg002_purge.$HAP.paf.gz |grep -v chrUn |grep -v alt |grep $tigID |head -n 1 |awk
'{print $2}'`

# skip contigs mapping to multiple chromosomes (mis-assemblies)
iserror=`cat tmp.bed |grep -c ERROR`
if [ $iserror -ne 0 ]; then
   echo "Cannot evaluate $tigID it maps to multiple chromosomes"
   continue
fi

# skip contigs w/o mappings to reference
iserror=`wc -l tmp.bed |awk ‘{print $1}’`
if [ $iserror -eq 0 ]; then
   echo “Error: cannot evaluate $tigID, no mappings”
   continue
fi

lenMapped=`cat tmp.bed |awk '{print $NF-$(NF-1)}'`
iserror=`echo "$len $lenMapped" |awk '{if ($1 / $2 > 0.5 && $1 / $2 < 2.0) print 1; else print
0}'`
if [ $iserror -eq 0 ]; then
   echo "Error: cannot evaluate $tigID it maps to $lenMapped and is only $len length, too
different"
   continue
fi

# extract regions from call sets that are TP and standardize on PAT/MAT
tabix -R tmp.bed calls.vcf.gz |grep "CALL=TP" |awk '{print $1"_"$2"\t"$2"\t"$4"\t"$5"\t"$NF}' |awk
'{if (match($NF, "0\\|1")) {print $0;} else if (match($NF, "1\\|0")) {print
$1"\t"$2"\t"$4"\t"$3"\t"$NF}}' > tmp

# extract regions from baseline which differ between mat and pat and standardize them to be
PAT/MAT
tabix -R tmp.bed baseline.vcf.gz |awk '{print $1"_"$2"\t"$2"\t"$4"\t"$5"\t"$NF}' |awk '{if
(match($NF, "0\\|1")) {print $0;} else if (match($NF, "1\\|0")) {print
$1"\t"$2"\t"$4"\t"$3"\t"$NF}}' |grep PATMAT > tmp2

num1=`wc -l tmp |awk '{print $1}'`
num2=`wc -l tmp2 |awk '{print $1}'`
if [ $num1 -eq 0 ]  || [ $num2 -eq 0 ]; then
   echo "Error: no snps in $tigID"
   continue
fi
java SubFile tmp2 tmp > tmp3 2>/dev/null
java SubFile tmp tmp2 > tmp4 2>/dev/null

# finally join and report when the variants are swapped (calling maternal) or matching (calling
paternal), we know they are the same set of variants since we’re only looking at true positives∂
```

```
join tmp3 tmp4 |awk -v CHR=$tigID -v LEN=$len -v PAT=0 -v MAT=0 '{if (!match($3, ",") &&
!match($4, ",") && !match($7, ",") && !match($8, ",")) { if  ($3 != $7 || $4 != $8) MAT+=1; else
PAT+=1;}} END {print CHR"\t"LEN"\t"PAT"\t"MAT}'
```

We ignore any contig without variants, any contig mapping to multiple chromosomes, any contig with too large or too small a mapping relative to its length, and any contig without reference mappings. Any variants not called as heterozygous were excluded from the assembly and the truth set. As expected, the primary contigs in the HiCanu assembly are pseudo-haplotypes and thus have an almost even split (44.61% switch error) between haplotypes. The alts are largely phased, with a switch rate of 5.15%. This is similar to the HiFi + HiC phased assembly (Garg et al. 2019) (4.61%/4.65%). The other assemblies could not be evaluated as they had a very low fraction of regions with variants called (< 100 Mbp alts).

Supplementary Figures

Supplementary Figure 1. *D. melanogaster* ISO1xA4 genomescope.

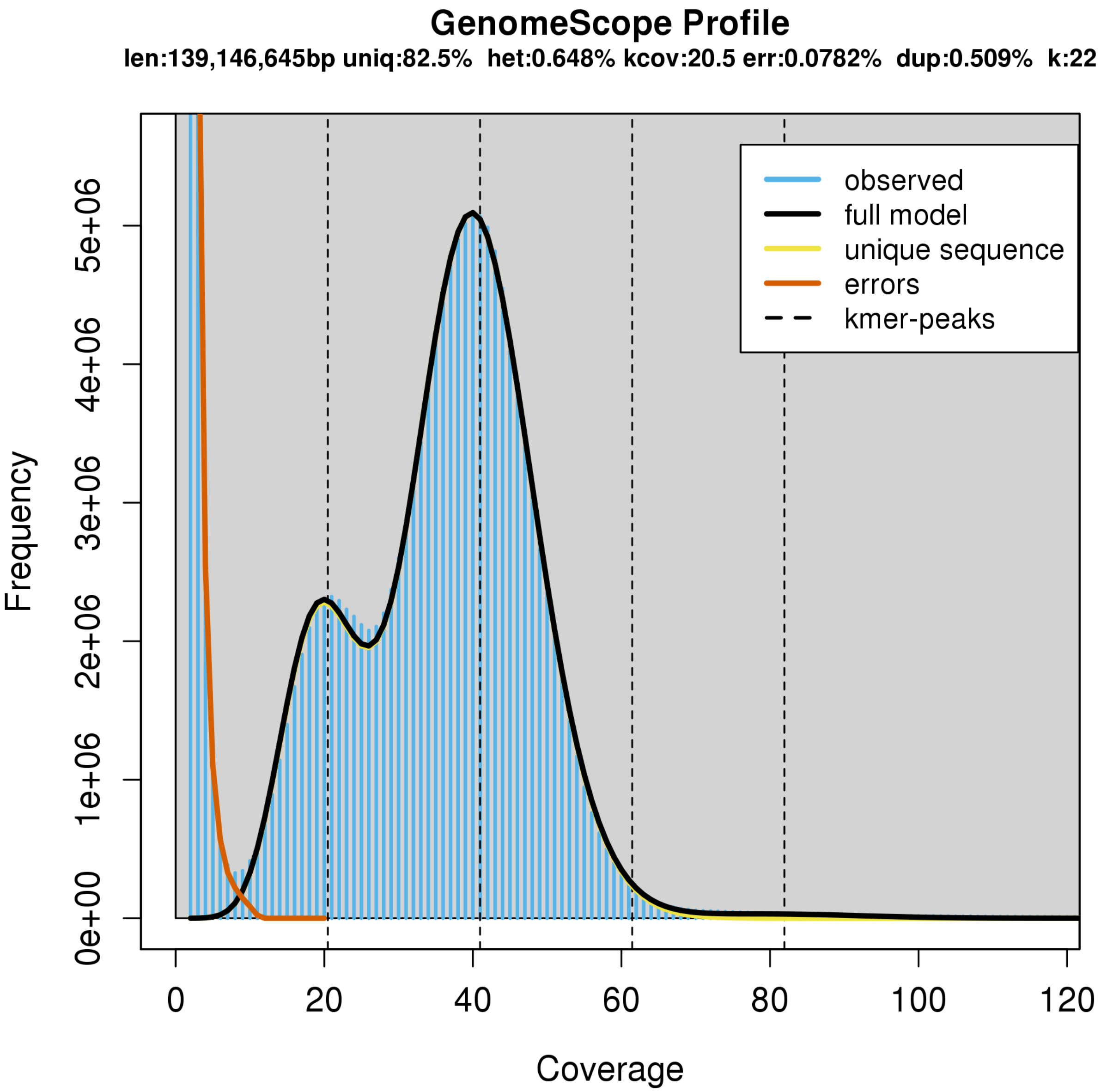

Estimate of genome size and heterozygosity using 22-mers found in the HiFi 24 kbp 40x down-sampled library for *D. melanogaster* ISO1xA4

Supplementary Figure 2. Evaluation of BAC AC279108.1.

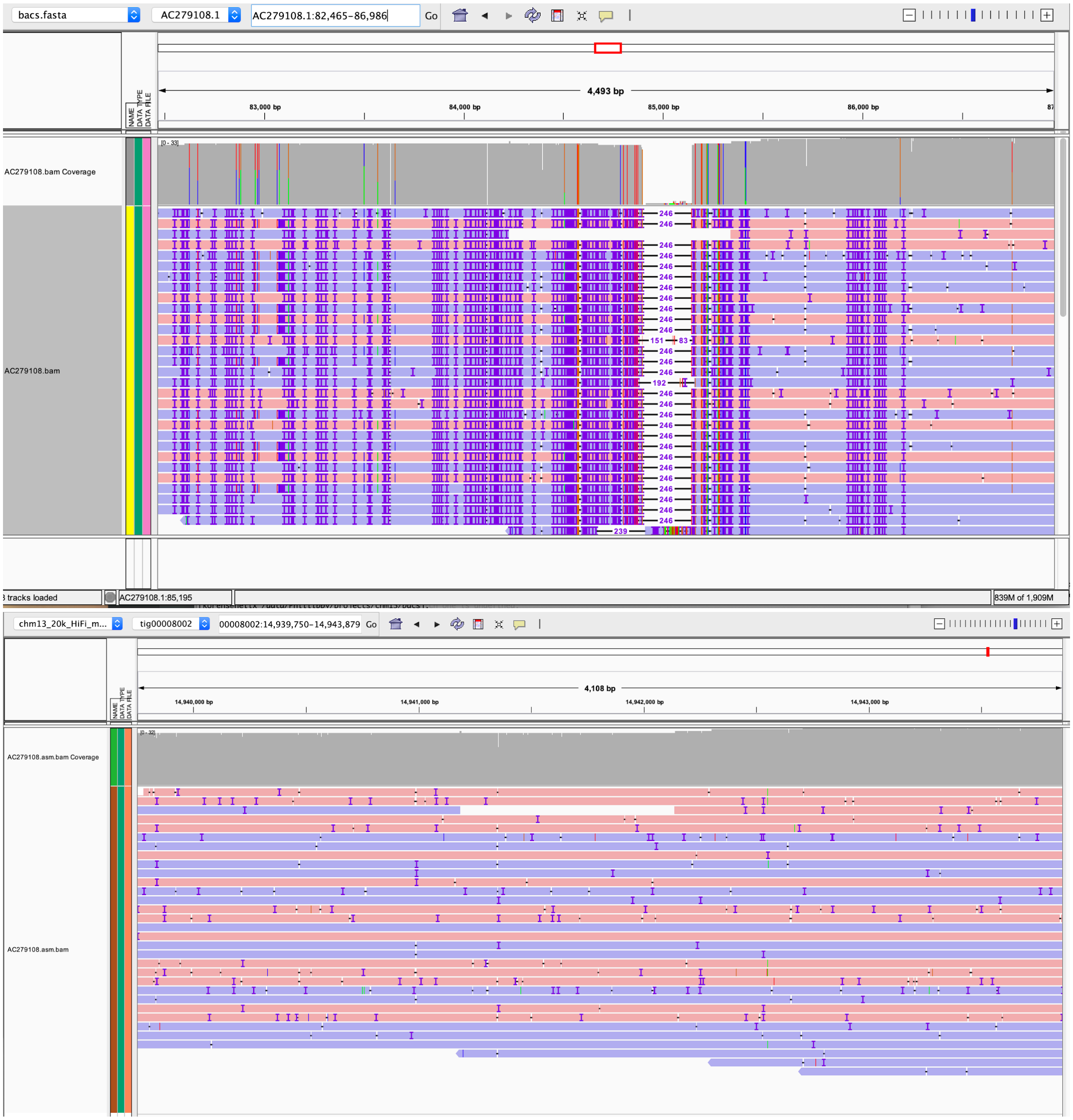

Top panel shows alignments of HiFi reads to the BAC sequence, bottom -- to the assembly. The top panel indicates a large SV called by the reads versus the BAC while the assembly is consistent with the data.

Supplementary Figure 3. Evaluation of BAC AC278968.1.

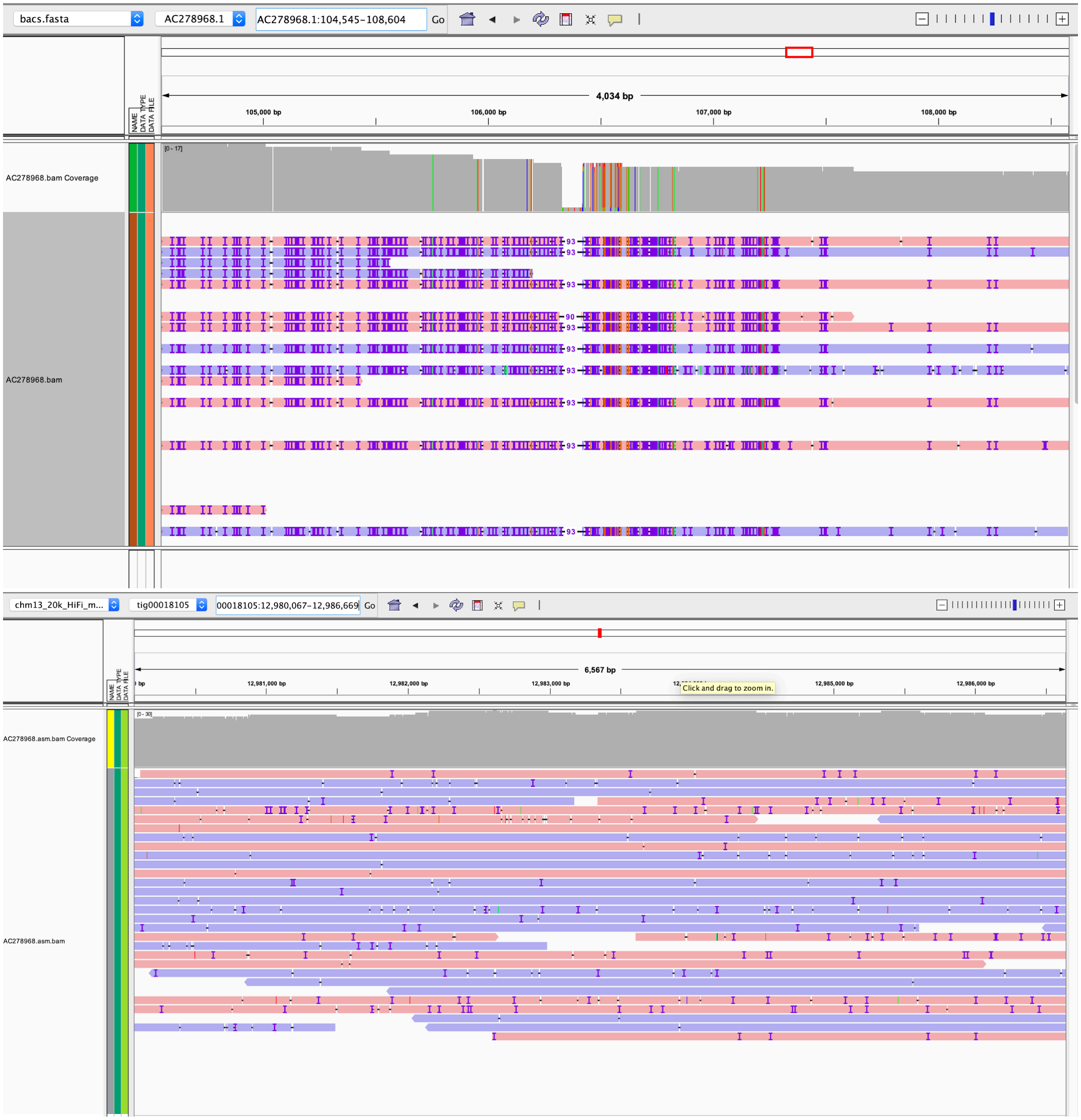

Top panel shows alignments of HiFi reads to the BAC sequence, bottom -- to the assembly. The top panel indicates a large SV called by the reads versus the BAC while the assembly is consistent with the data.

Supplementary Figure 4. Evaluation of BAC AC278708.1.

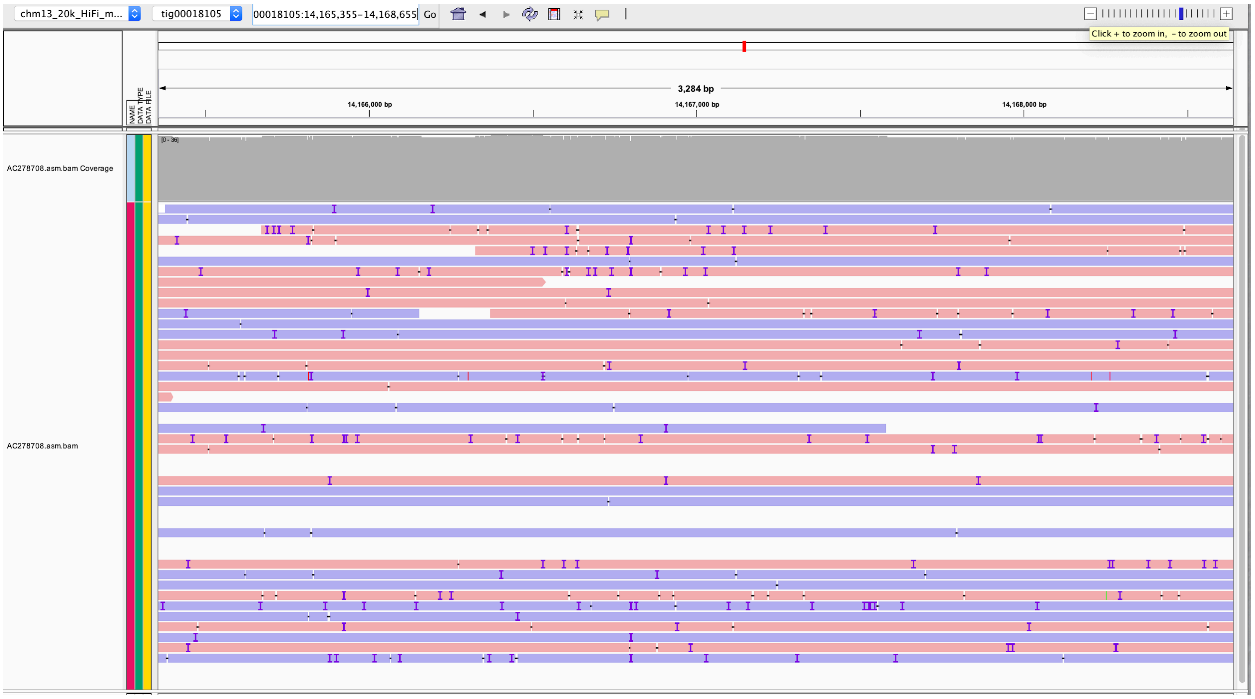

Alignments of HiFi reads to the corresponding assembly region, differing from the BAC sequence. No apparent SVs, SNVs, or coverage anomalies are visible. Read alignments to the BAC sequence are not shown as the breakpoint region is at the end of the BAC and thus has a coverage drop due to edge effects. The HiCanu assembled version is further supported by GRCh38.p13.

Supplementary Figure 5. Evaluation of BAC AC278985.1.

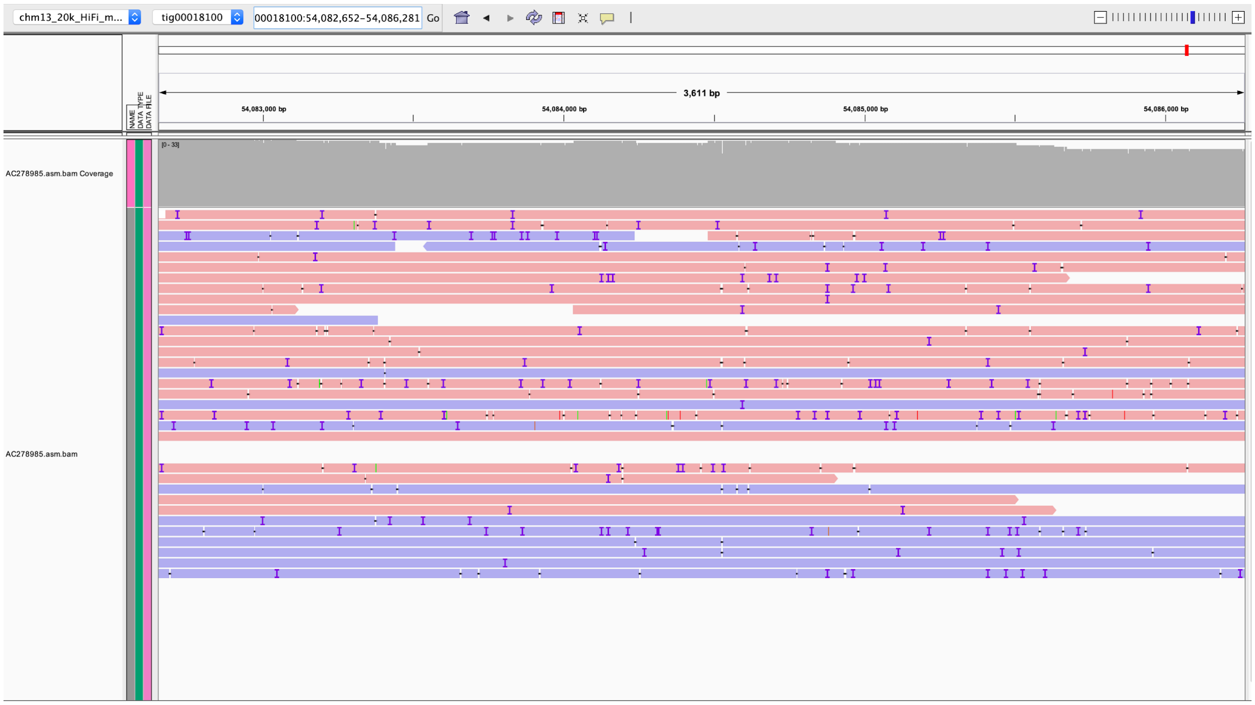

Alignments of HiFi reads to the assembly region, differing from the BAC sequence. No apparent SVs, SNV, or coverage anomalies are visible. Read alignments to the BAC sequence are not show as the breakpoint region is at the end of the BAC and thus has a coverage drop due to edge effects. The HiCanu assembled version is further supported by GCRh38.p13.

Supplementary Figure 6. Unresolved repeats by class in CHM13 assemblies.

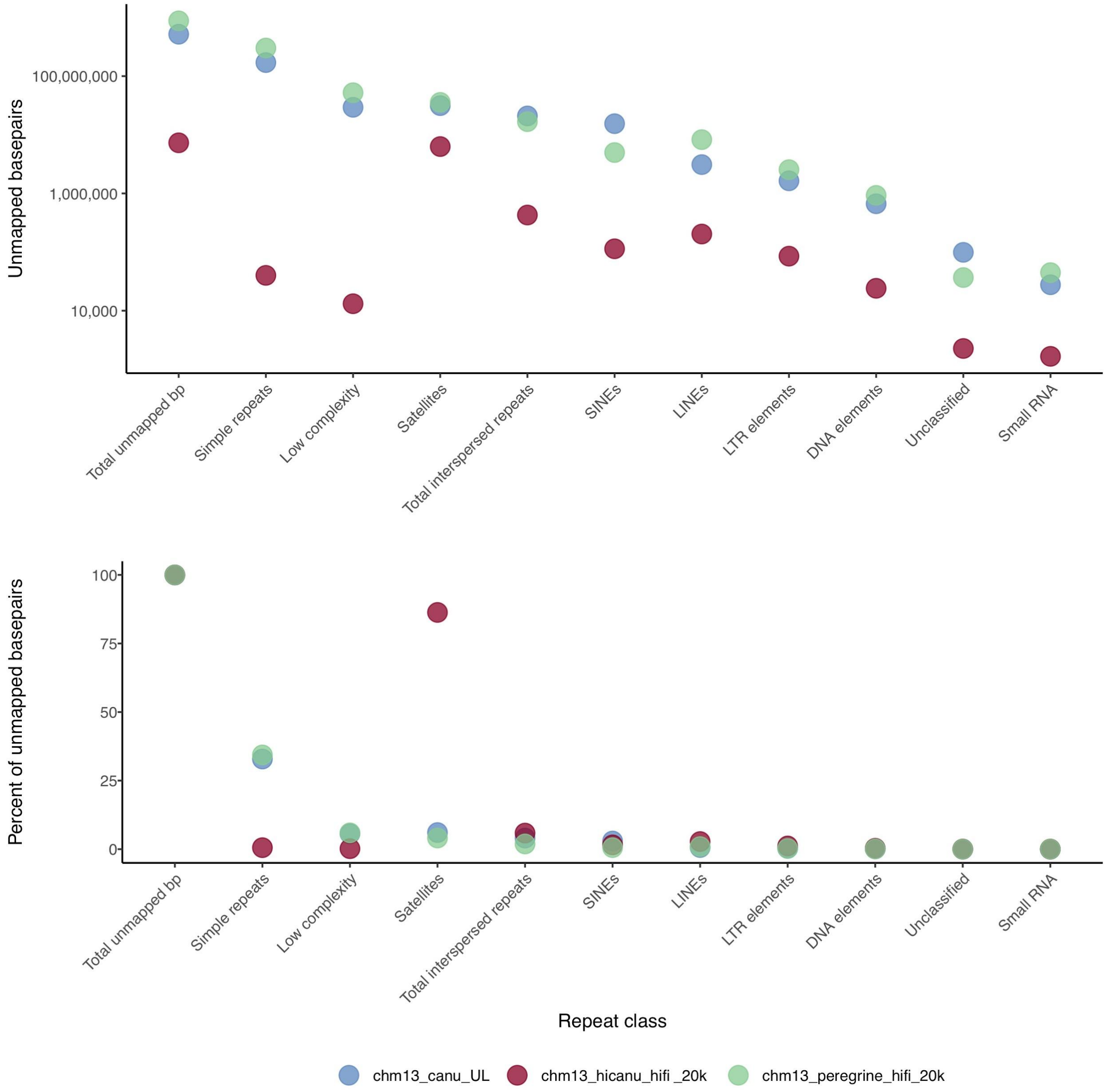

The total number of bases in unmapped reads (top, log-scaled) and their fraction (bottom) in three assemblies of CHM13. All HiFi reads were aligned to the assembly with minimap2 v2.17 (`minimap2 -ax asm20 --secondary=no -s 4000 {input.reads} | samtools view -b - > {output.bam}`), unmapped reads converted to fasta (`samtools fasta -f 4 {output.bam} > {output.fasta}`), and repeats identified by RepeatMasker (`RepeatMasker -species human -e wublast -dir  {ouput.dir} {output.fasta}`). HiCanu (red) improves over both peregrine (green) and ONT UL (blue) assemblies in all repeat types, most notably in simple repeats and low complexity sequences. The majority of remaining unrepresented sequences (over 80% of total) in HiCanu are satellite repeats.

Supplementary Figure 7. Predicted human assembly continuity based on sequencing read length and accuracy.

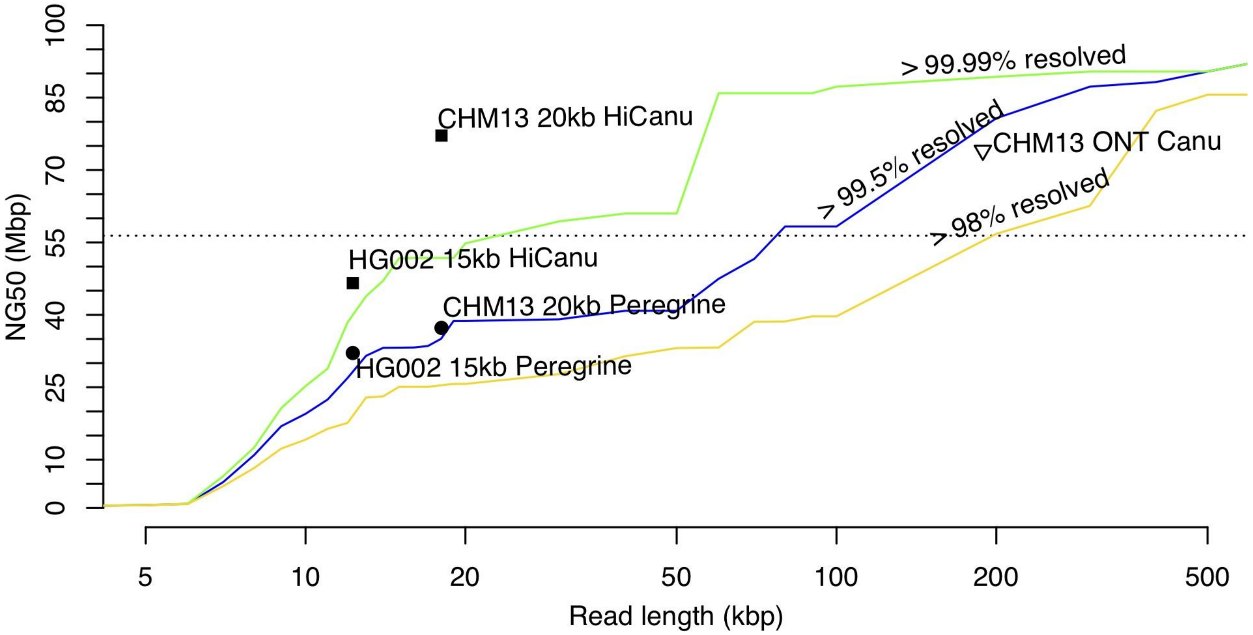

Assembly continuity (NG50 contig size) is dependent on read length (x-axis) and read accuracy (colored curves). Model curves are plotted for three hypothetical assemblers able to separate repeats with 98%, 99.5%, and 99.99% average sequence identity. Points represent real assemblies from Peregrine HiFi (Chin and Khalak 2019), HiCanu HiFi (this work), and Canu Nanopore (Miga et al. 2020). The dashed line corresponds to the continuity of human reference genome GRCh38 (Schneider et al. 2017). Real assemblies typically perform better than predicted by the model, due to the unknown divergence level of many large human repeats which are considered as perfect repeats within the model. In reality, many of the repeat instances appear to have enough variants to be effectively resolved by the assembler.

Supplementary Figure 8. HiFi mapping coverage of HiCanu centromere assemblies

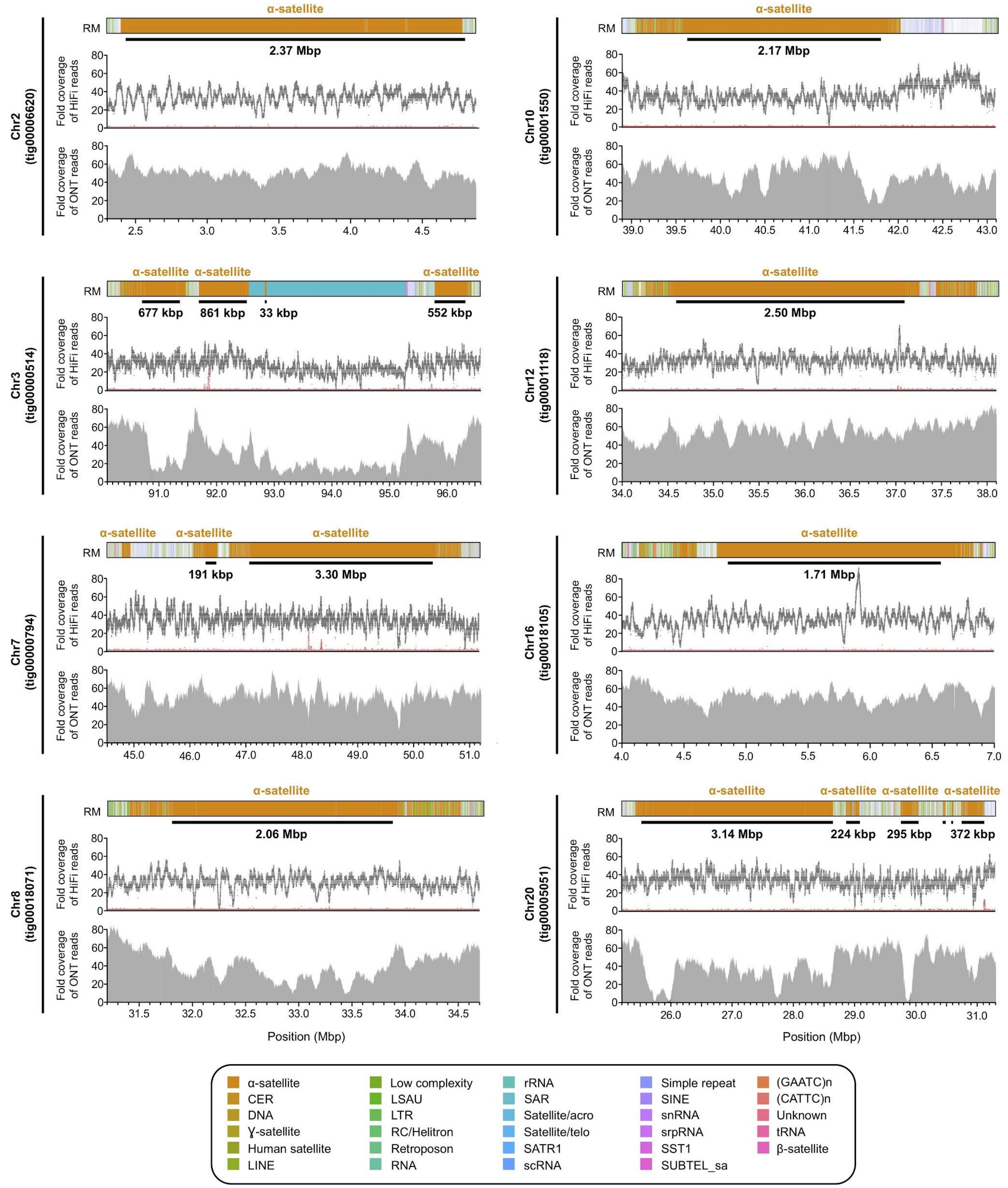

In addition to Chromosome 19, HiCanu produced draft assemblies for eight other centromeric satellite arrays of the CHM13 genome. RepeatMasker (RM) annotation reveals the location of α-satellite HOR arrays (marked with a black bar) in each contig. Alignment of HiFi data to each contig reveals even coverage, except for rare dips and spikes in coverage within contigs from Chromosomes 8, 10, 12, and 16, which may indicate mis-mapping, mis-assembly, and/or collapse in sequence. Further validation is required to determine the accuracy of these centromeric assemblies.

Supplementary Figure 9. Sequence identity between the HiCanu D19Z1 or D19Z3 α-satellite arrays and the D19Z1 or D19Z3 α-satellite sequences.

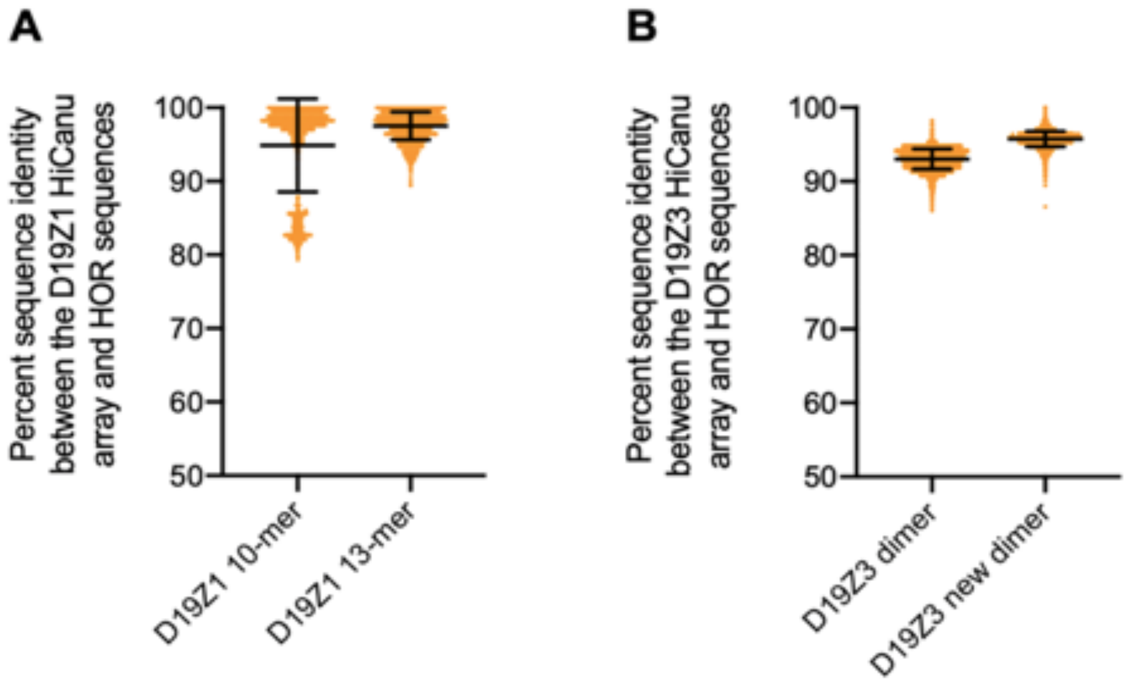

A) Plot showing the sequence identity of each α-satellite repeat within the HiCanu D19Z1 array and the previously identified D19Z1 10-mer (left; Hulsebos et al. 1988; Puechberty et al. 1999; pG-A16, AJ295045.1) or the newly identified D19Z1 13-mer (right). The cluster of low-sequence-identity α-satellite repeats on the left is a result of poor sequence alignment to the 10-mer. When the three newly-identified α-satellite repeats are included to form the 13-mer, this cluster has increased sequence identity, and the overall mean sequence identity increases (94.87 +/- 6.32% vs. 97.51 +/- 1.91%). B) Plot showing the sequence identity of each α-satellite repeat within the HiCanu D19Z3 array and the previously identified D19Z3 dimer (left; Baldini et al. 1989; pC1.8, M26919 and M26920) or the newly identified D19Z3 dimer (right). Almost all α-satellite repeats within the HiCanu D19Z3 array have higher sequence identity to the newly identified dimer than to the previously published dimer (95.74 +/- 1.05% vs. 93.02 +/- 1.37%). Mean +/- SD is shown.

Supplementary Figure 10. Chromosome 19 D19Z1, D19Z2, and D19Z3 α-satellite HOR array structures.

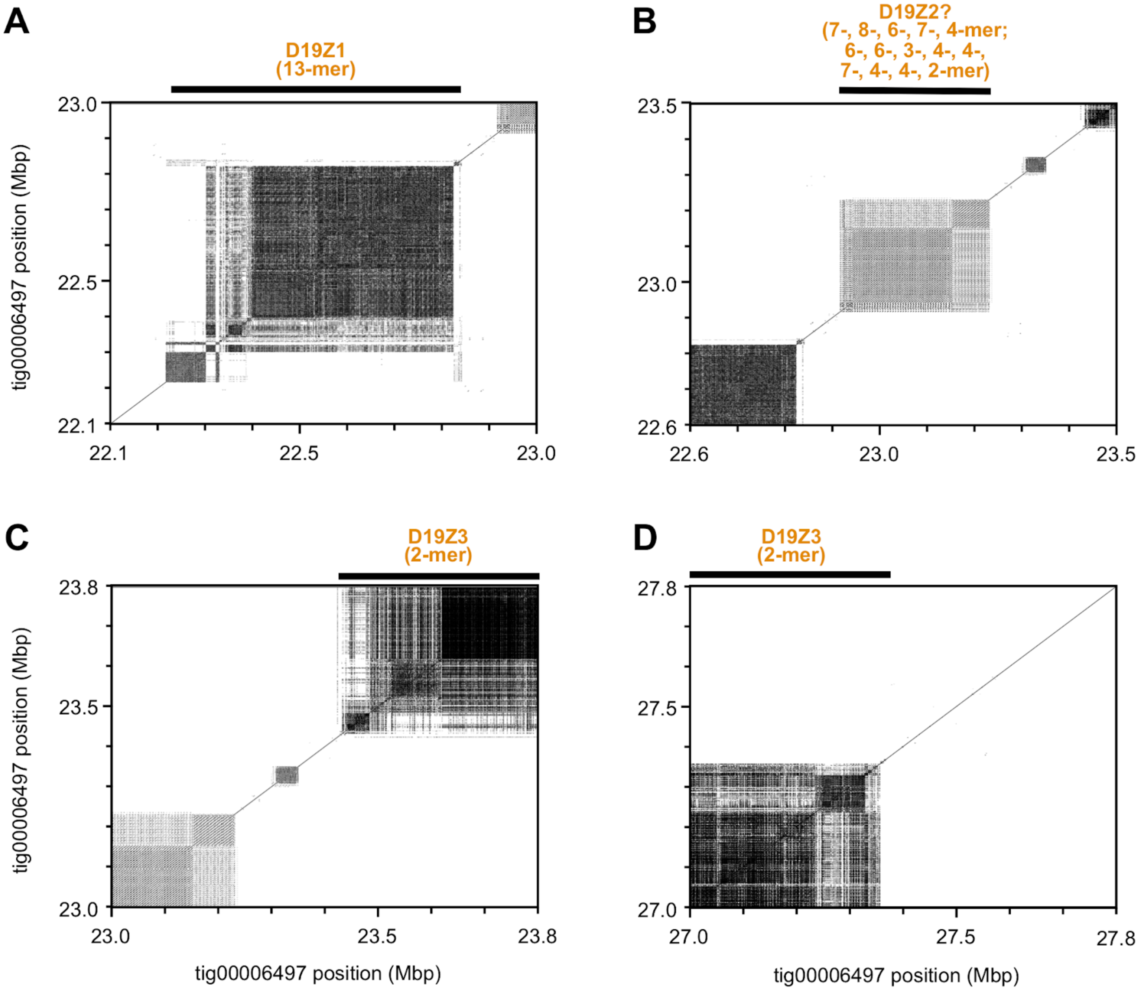

Dot plots showing the high sequence identity and structure of the D19Z1 array (A), D19Z2 array (B), and edges of the D19Z3 array (C, D). The D19Z2 array has two complex HOR structures that can be observed in panel B (see Main Text for details). Because the HOR array structures in this region do not match the expected pG-A16 repeat structure, we designate it here as 'D19Z2?'. Dot plots were generated with a word length of 100.

Supplementary Figure 11. Segmental duplications associated with contig ends.

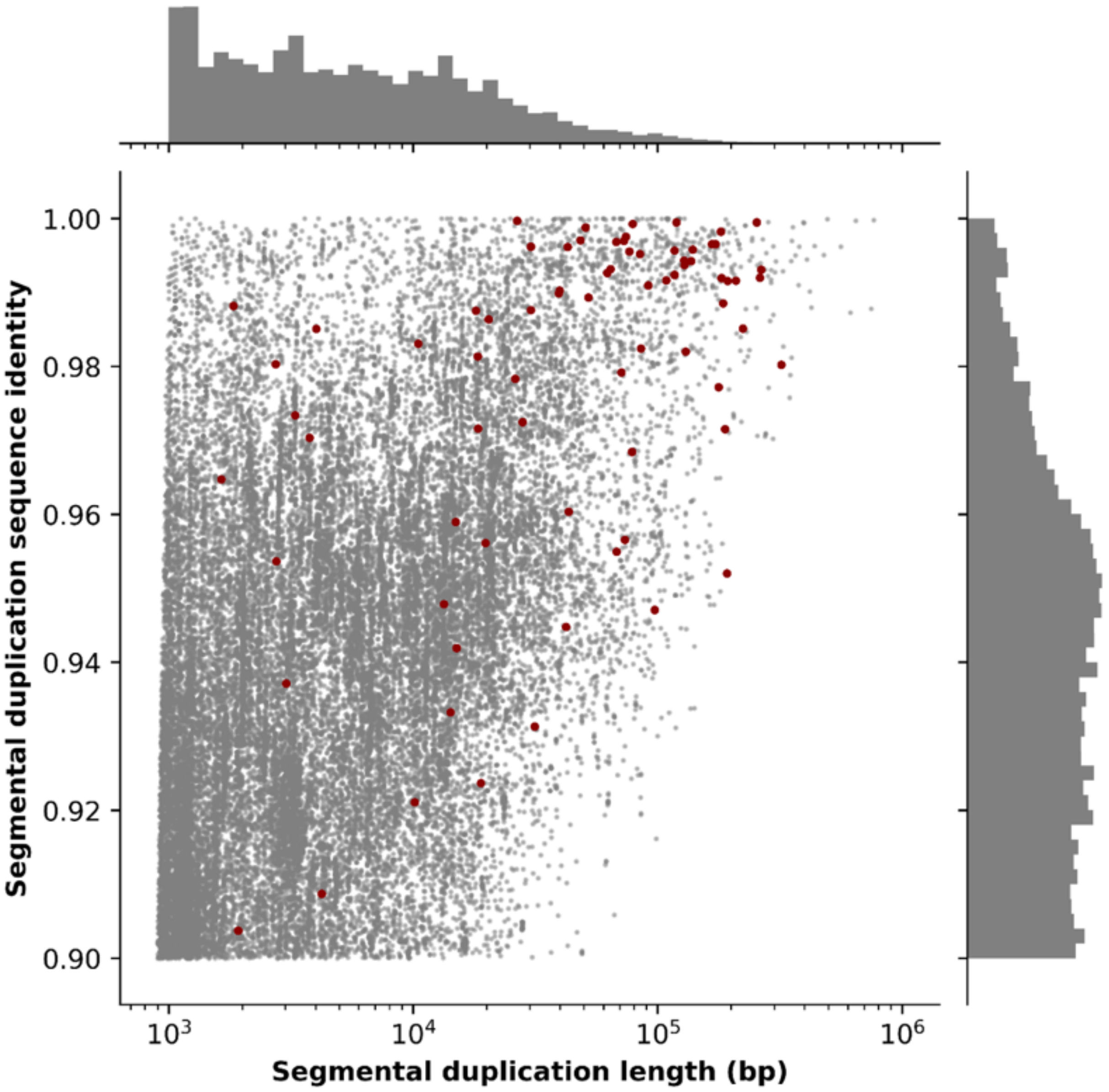

Shown are the sequence identity and length of all segmental duplications in GRCh38 (gray). Red data points indicate the largest segmental duplication within 10 kbp of the 95 contig ends located within segmental duplications in the CHM13 20 kbp HiCanu assembly.

Supplementary Figure 12. Coverage drop in defensin reference.

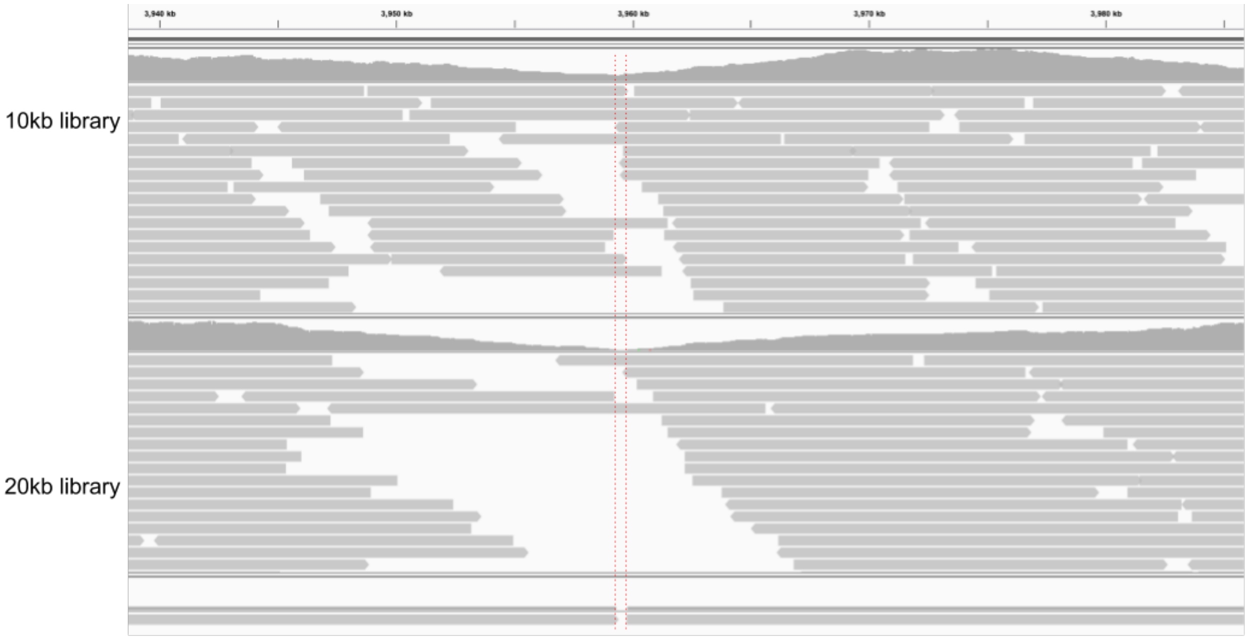

Contig ‘break’ in the HiCanu 20 kbp assembly (highlighted in red) corresponds to a coverage drop in both the 10 kbp and 20 kbp HiFi library. The region immediately upstream of the gap contains a >200 bp (AAAGG) simple-sequence repeat.

Supplementary Figure 13. ChrX assembly comparison.

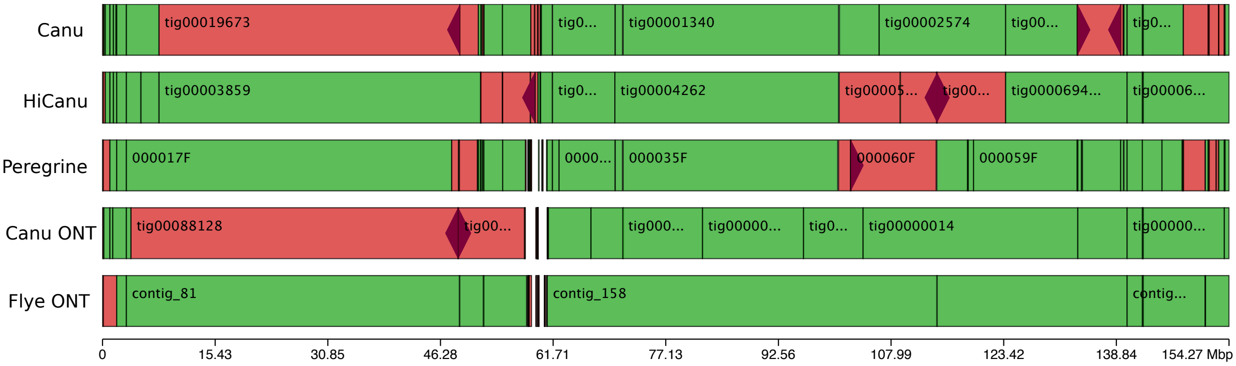

Icarus (Mikheenko et al. 2016) visualization of contig alignments from both HiFi-based (Canu, HiCanu, Peregrine) and ultra-long Nanopore-based assemblies (Canu ONT and Flye ONT (Kolmogorov et al. 2019)) produced by QUAST (Gurevich et al. 2013), ignoring local mis-assemblies (< 5 kbp), against the T2T ChrX reference (Miga et al. 2020). White space in the alignment figure indicates the assembly was fragmented into short contigs (<50 kbp). Blocks show contig alignments split at QUAST mis-assembly boundaries (both local and extensive as indicated by vertical lines). Blocks ending in an extensive mis-assembly are colored red. The ONT assemblies are more continuous with chromosome-arm scale contigs: Canu ONT 82.7 Mbp and Flye 90.6 NG50. All HiFi assemblies are less continuous, with HiCanu having the largest NG50 (30.6 Mbp), Canu HiFi (20.3 Mbp), and Peregrine (14.2 Mbp). HiCanu has the lowest rate of extensive mis-assemblies (5) versus Canu HiFi (11), Peregrine (10), Canu ONT (32), and Flye (14). HiCanu also has the most continuous reconstruction of the CENX array, splitting it across two contigs. Canu HiFi has the CENX array in three contigs while the other assemblers have no long contigs which can be aligned to the centromere.

Supplementary Figure 14. Read alignment identity against ChrX

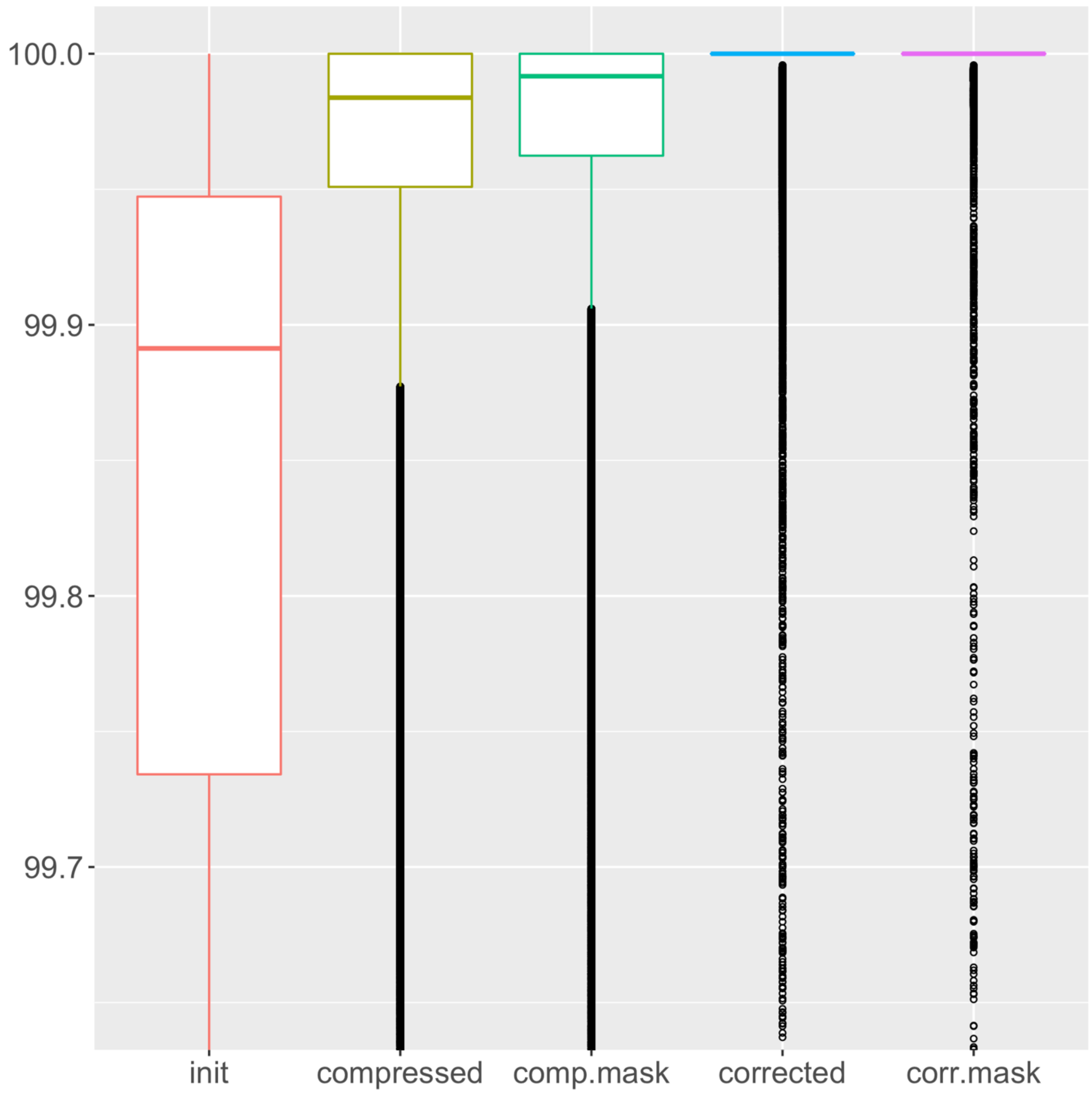

Separate boxplots are shown for raw HiFi reads as in Figure 1 (init), homopolymer-compressed reads (compressed), OEA-corrected reads (corrected), and corrected reads after ignoring differences in microsatellite repeats (masked). This figure also includes compressed + masked reads to evaluate the impact of our correction. The median read identity, indicated by solid segments, increases from less than 99.9% to 100% (note that the plots show an y-range of 99.65–100%). Corrected + masked reads are comparable to just compressed reads and correction is necessary to achieve the near-perfect read alignments.

Supplementary Figure 15. Bubble contig analysis in HiCanu.

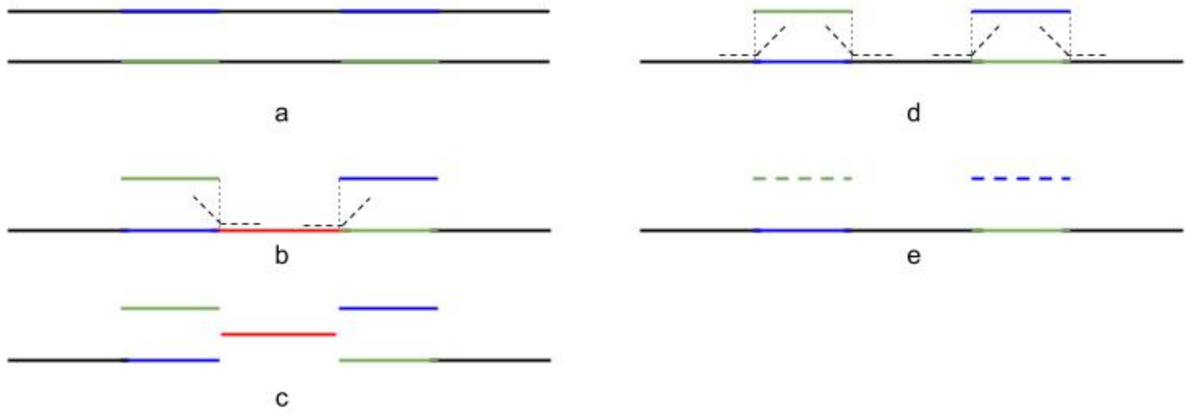

a. Genomic area, containing two regions of relatively high heterozygosity (blue/green) divided by a long homozygous region (black). **b.** The homozygous region is typically classified as a genomic repeat due to the reads at boundaries between homozygous and flanking heterozygous regions. **c.** The large pseudo-haplotype contig is broken at the region boundaries. **d.** In HiCanu, bubble contigs (see main text for the definition) are first detected and the reads crossing the regions boundary are no longer considered in repeat detection. **e.** The pseudo-haplotype contig is not split, leaving a continuous pseudo-haplotype.

Supplementary Tables

Supplementary Table 1. Alignment-based read identity stats.

	raw reads	compressed	Compressed + Microsatellite mask	OEA-corrected	OEA-corrected + Microsatellite mask
Total	262 664	263 526	263 559	263 529	263 559
=100% identity	907	93 840	119 161	206 711	256 259
<100% identity	261 757	169 686	144 398	56 818	7 300
Percent with 100% identity alignment	0.35%	35.61%	45.21%	78.44%	97.23%

The analysis used 20 kbp HiFi CHM13 library. All reads were aligned to the recently finished ChrX reference. Reads were output from HiCanu after compression, after compression and ignoring errors within microsatellite regions, after OEA-correction, and all steps combined (compression + OEA-correction + microsatellite mask).

Supplementary Table 2. BUSCO statistics and Illumina QV (*k*=18 and *k*=31) QV for *D. melanogaster* assemblies.

Assembly	Size (Mbp)	NG50 (Mbp)	Complete BUSCOs	Duplicated BUSCOs	*k*=18 QV	*k*=31 QV
Canu CLR	293.73	15.20	98.8%	84.7%	37.4<br>35.5	36.6<br>35.9
Peregrine	162.41	12.68	98.1%	1.1%	32.9<br>33.5	33.9<br>30.8
Canu	292.83	13.72	99.0%	92.0%	51.9<br>46.9	47.6<br>44.6
HiCanu	300.93	20.16	98.9%	94.6%	51.0<br>46.7	47.1<br>44.5

A genome size of 143,726,002 was used for NG50 computation. We report Illumina-based QV both for *k*=18 (as in Table 1) and *k*=31. Only contigs >=50 kbp were included for analysis.

Supplementary Table 3. QUAST and QV results for human assemblies.

Genome	Assembly	NGA50 (Mbp)	# diffs	# diffs outside known SVs/centromere/segdups	QV (*k*=31)
CHM13	Canu ONT	**23.83**	5,179	**105**	27.1
	Canu	18.96	14,501	123	**57.4**
	Peregrine	21.59	3,017	117	48.3
	HiCanu	21.72	15,148	140	55.2
HG00733	Canu ONT	15.92	2,632	198	22.8<br>22.2
	Haplotype asm hap1	15.77	3,551	165	46.4
	Haplotype asm hap2	15.77	3,463	143	46.4
	Canu	15.14	22,144	252	46.7<br>40.0
	Peregrine	**21.93**	3,416	215	43.5<br>32.4
	HiCanu	20.51	27,721	304	**46.8**<br>**41.7**
	HiCanu (primary)	16.85	13,787	**93**	**46.8**
HG002	Canu ONT	15.79	2,320	171	22.0<br>21.8
	Haplotype asm hap1	14.33	5,854	355	45.0
	Haplotype asm hap2	13.50	5,370	343	44.8
	Canu	17.01	15,120	156	32.6<br>42.4
	Peregrine	16.52	2,993	102	44.5<br>32.6
	HiCanu	**21.72**	21,672	229	**49.2**<br>**43.4**
	HiCanu (primary)	17.65	12,019	**73**	**49.2**

NGA50s used 3,098,794,149 as genome size. GRCh38 excluding unassigned and alts contigs was used as reference. "# diffs" is the sum of relocations, translocations, and inversions from QUAST v5.0.2. Using scripts from Safin et al., we ignored differences within known variants (for HG002 only) and centromeres and segmental duplications since they are likely enriched for real differences between the reference and sequenced genomes. Haplotype-resolved assemblies are from Garg et al. 2019 (HG002) and Porubsky et al. 2019 (HG00733). QV results were computed using Merqury with *k*=31 and include only contigs >= 50 kbp.

Supplementary Table 4: CHM13 10 kbp HiFi assemblies

Assembly	NG50 Mbp	Total Gbp	Illumina QV (*k*=21)	Illumina QV (*k*=31)	BACs	BAC QV	NGA50 (Mbp)	# QUAST diffs	# diffs outside contigs <50kb, known SVs/centromere/segdups	Complete BUSCOs	Duplicated BUSCOs
Canu + Racon x2	29.1	2.94	**61.8**	57.2	155/341	36.9	17.39	6,809	112	94.8%	1.3%
Peregrine	29.7	2.83	51.3	49.0	122/341	34.8	16.87	**1,219**	**97**	94.7%	**1.1%**
HiCanu	**39.3**	3.03	61.7	**57.3**	**309/341**	**40.8**	**19.24**	12,001	114	**95.1%**	1.4%

CHM13 10 kbp assemblies from Canu, Peregrine, and HiCanu were evaluated as before. All statistics are reported on contigs >= 50 kbp.

Supplementary Table 5. HiC+HiFi and StrandSeq+HiFi published assemblies

Genome	Assembly	Size (Gbp)	NG50 (Mbp)	Quality (QV)	QUAST (diffs per Gbp)	#BACs resolved	BAC Idy
HG002	HiC+HiFi[a]	2.86<br>2.86	22.87<br>23.03	46.4<br>46.1	124.1<br>120.0	N/A	N/A
HG00733	StrandSeq+HiFi[b]	2.86<br>2.86	28.46<br>23.62	50.5<br>50.4	57.7<br>50.0	84/179	28.1

A genome size of 3,098,794,149 was used for computing NG statistics. Only contigs ≥ 50 kbp were used for all analyses except QUAST. Structural differences in centromeric regions and segmental duplications were ignored due to instability in these regions, and diffs were normalized by assembly size (in Gbp). Superscripts mark previously published assemblies: [a]Garg et al. 2019, [b]Porubsky et al. 2019. The criteria for considering a BAC "resolved" is described in the main text and Methods. We combined both haplotypes and counted a BAC as resolved if either haplotyped resolved it. The alignment identity of each resolved BAC was computed individually and the median of these values reported as a Phred-style quality value. No validation BACs were available for HG002.

Supplementary Table 6. CHM13 corrected BACs results.

Genome	Assembly	Original BACs resolved	Corrected BACs resolved
CHM13	Canu ONT (Miga et al. 2020)	314/341	323/341
	Canu 10kb	155/341	157/341
	Canu 20kb	308/341	316/341
	Peregrine 10kb	122/341	124/341
	Peregrine 20kb	136/341	137/341
	HiCanu 10kb	309/341	320/341
	HiCanu 20kb	326/341	**337/341**

CHM13 BACs were downloaded from the library VMRC59 at NCBI. BACs putatively identified as incorrect were replaced by their reconstruction from the HiCanu 20 kbp assembly. Statistics were computed using the scripts at https://github.com/skoren/bacValidation. Despite the BAC corrections coming from the assembly of the 20kbp HiFi dataset, 8 were resolved by at least two other assemblies (Peregrine 10 kbp/20 kbp, Canu 10 kbp/20 kbp/Canu UL), and three were resolved in one other assembly (AC278859.1 Canu ONT, AC278985.1 Canu ONT, AC279108.1 Canu 10 kbp), supporting the updated sequences.

Supplementary Table 7. CHM13 collapsed bases.

Datatype	Assembly	Collapsed (Mbp)	Expanded (Mbp)
ONT UL	Canu ONT	35.00	254.36
HiFi 10kbp	Canu + Racon x2	53.74	183.60
	Peregrine	45.57	328.42
	HiCanu	27.41	78.56
HiFi 20kbp	Canu	24.88	71.03
	Peregrine	44.85	309.27
	HiCanu	**22.80**	**56.73**

CHM13 collapses were evaluated using SDA as in Vollger et al. 2019. All contigs in the assembly are included for analysis. The *collapsed* bases are the assembly bases with “higher than expected coverage, while the *expanded* bases is the estimate of how much sequence is contained in the collapsed regions, had they been correctly assembled.

Supplementary Table 8. BUSCO and phase block statistics.

Genome	Assembly	Complete BUSCOs	Duplicated BUSCOs	Phase block NG50 (Mbp)	Intra-block switch rate
CHM13	Canu ONT	79.3%	1.0%	N/A	N/A
	Canu	94.7%	1.5%	N/A	N/A
	Peregrine	94.8%	1.4%	N/A	N/A
	HiCanu	94.1%	2.7%	N/A	N/A
HG00733	Canu ONT (alts)	66.3% (0.3%)	0.8% (0.0%)	0.23 (0.00)	18.27 (11.74)
	Haplotype asm hap1 (hap2)[a]	94.8% (95.2%)	1.4% (1.4%)	5.14 (4.49)	0.43 (0.56)
	Canu primary (alts)	94.8% (22.7%)	1.5% (1.2%)	0.17 (0.00)	5.20 (0.12)
	Peregrine (alts)	95.0% (0.5%)	1.3% (0.1%)	0.15 (0.00)	8.47 (5.01)
	HiCanu primary (alts)	94.9% (77.2%)	1.4% (1.9%)	0.62 (0.14)	0.45 (0.12)
HG002	Canu ONT (alts)	63.4% (0.2%)	0.8% (0.0%)	0.23 (0.00)	11.24 (0.97)
	Haplotype asm hap1 (hap2)[b]	94.7% (95.0%)	1.2% (1.2%)	12.82 (11.42)	0.13 (0.13)
	Canu primary (alts)	92.1% (5.5%)	1.2% (0.3%)	0.15 (0.00)	3.18 (0.07)
	Peregrine (alts)	95.1% (0.6%)	1.2% (0.1%)	0.15 (0.00)	5.43 (0.30)
	HiCanu primary (alts)	94.8% (77.1%)	1.2% (1.7%)	0.65 (0.10)	0.24 (0.01)

All contigs were included for analysis, regardless of length. [a]Assembly generated in Porubsky et al. 2019. [b]Assembly generated in Garg et al. 2019.

Supplementary Table 9. GIAB variant analysis on HG002.

Assembly	Sensitivity	Precision	F1	Merqury maternal complete	Merqury paternal complete	Merqury average complete
Canu ONT	30.28%	5.88%	9.85%	41.21%	53.30%	47.26%
Haplotype asm[a]	**98.14%**	**97.28%**	**97.70%**	97.50%	97.19%	97.35%
Canu	37.10%	30.87%	33.70%	60.14%	72.56%	66.35%
Peregrine	37.48%	53.56%	44.10%	51.17%	64.47%	58.32%
HiCanu	96.11%	94.65%	95.37%	**98.15%**	**97.84%**	**98.00%**

All binned primary and alternate assembly contigs were included for analysis, regardless of length. Variants were identified by dipcall and statistics measured by vcfeval against GIAB v3.3.2 trio-phased variant calls. We also measured maternal and paternal haplotype completeness using Merqury which agreed well with the gold-standard variant calls. [a]Assembly generated in Garg et al. 2019.

Supplementary Table 10. HLA gene truth typing from Chin et al. 2019 (HG002) and Shafin et al. 2020 (HG00733).

Genome	Gene	Expected haplotype1	Expected haplotype2
HG00733	HLA-A	30:02:01G	24:02:01G
	HLA-B	18:01:01G	35:02:01G
	HLA-C	05:01:01G	04:01:01G
	HLA-DQA1	05:01:01G	05:01:01G
	HLA-DQB1	02:01:01G	03:01:01G
	HLA-DRB1	03:01:01G	11:04:01G
HG002	HLA-A	26:01:01G	01:01:01G
	HLA-B	38:01:01G	35:08:01G
	HLA-C	12:03:01G	04:01:01G
	HLA-DQA1	03:01:01G	01:01:01G
	HLA-DQB1	03:02:01G	05:01:01G
	HLA-DRB1	04:02:01G	10:01:01G

Supplementary Table 11. HLA gene typing results across different assemblies.

Genome	Assembly	Contig	Gene	Called	Edit Distance
HG0733	Canu	tig00032730	HLA-A	A*30:02:01G	4
		tig00032730	HLA-B	B*35:02:01G	0
		tig00032730	HLA-C	C*04:01:01G	0
		tig00062315	HLA-DQA1	DQA1*05:01:01G	0
		tig00062315	HLA-DQB1	DQB1*03:01:01G	0
		tig00062315	HLA-DRB1	DRB1*11:04:01G	0
		tig00037512	HLA-A	A*24:02:01G	0
		tig00037527	HLA-B	B*18:01:01G	0
		tig00037527	HLA-C	C*05:01:01G	0
		tig00038945	HLA-DQA1	DQA1*05:01:01G	0
		tig00038945	HLA-DQB1	DQB1*02:01:01G	0
		tig00038955	HLA-DRB1	DRB1*03:01:01G	0
	HiCanu	tig00018936	HLA-A	A*30:02:01G	0
		tig00018936	HLA-B	B*35:02:01G	0
		tig00018936	HLA-C	C*04:01:01G	0
		tig00023025	HLA-DQA1	DQA1*05:01:01G	0
		tig00023025	HLA-DQB1	DQB1*03:01:01G	0
		tig00023025	HLA-DRB1	DRB1*11:04:01G	0
		tig00029466	HLA-A	A*24:02:01G	0
		tig00029414	HLA-B	B*18:01:01G	0
		tig00029414	HLA-C	C*05:01:01G	0
		tig00023598	HLA-DQA1	DQA1*05:01:01G	0
		tig00023598	HLA-DQB1	DQB1*02:01:01G	0
		tig00023598	HLA-DRB1	DRB1*03:01:01G	0
	StrandSeq HiFi	000030F	HLA-A	A*30:02:01G	0

		000030F	HLA-B	B*35:02:01G	0
		000030F	HLA-C	C*04:01:01G	0
		000030F	HLA-DQA1	DQA1*05:01:01G	0
		000030F	HLA-DQB1	DQB1*03:01:01G	0
		000030F	HLA-DRB1	DRB1*11:04:01G	0
		000118F	HLA-A	A*30:02:01G	0
		000118F	HLA-B	B*35:02:01G	0
		000118F	HLA-C	C*05:01:01G	0
		000031F	HLA-DQA1	DQA1*05:01:01G	0
		000031F	HLA-DQB1	DQB1*02:01:01G	0
		000031F	HLA-DRB1	DRB1*03:01:01G	0
	Peregrine	000029F	HLA-A	A*30:02:01G	4
		000029F	HLA-B	B*35:02:01G	1
		000029F	HLA-C	C*04:103	3
		000029F	HLA-DQA1	DQA1*05:01:01G	0
		000029F	HLA-DQB1	DQB1*03:01:01G	0
		000029F	HLA-DRB1	DRB1*11:04:01[illegible]	0
HG002	Canu	tig00038929	HLA-A	A*01:01:01G	0
		tig00038929	HLA-B	B*38:01:01G	0
		tig00038929	HLA-C	C*04:01:01G	0
		tig00038927	HLA-DQA1	DQA1*03:01:01G	0
		tig00038927	HLA-DQB1	DQB1*03:02:01G	0
		tig00038927	HLA-DRB1	DRB1*04:02:01	0
		tig00013892	HLA-A	A*26:01:01G	0
		tig00013881	HLA-B	B*35:08:01G	0
		tig00013903	HLA-C	C*12:03:01G	0
		tig00007817	HLA-DQA1	DQA1*01:01:01G	0

		tig00007817	HLA-DQB1	DQB1*05:01:01G	0
		tig00007817	HLA-DRB1	DRB1*10:01:01G	0
	HiCanu	tig00009386	HLA-A	A*01:01:01G	0
		tig00009386	HLA-B	B*35:08:01G	0
		tig00009386	HLA-C	C*04:01:01G	0
		tig00009386	HLA-DQA1	DQA1*03:01:01G	0
		tig00009386	HLA-DQB1	DQB1*03:02:01G	0
		tig00009386	HLA-DRB1	DRB1*04:02:01	0
		tig00009431	HLA-A	A*26:01:01G	0
		tig00017533	HLA-B	B*38:01:01G	0
		tig00017533	HLA-C	C*12:03:01G	0
		tig00009488	HLA-DQA1	DQA1*01:01:01G	0
		tig00009488	HLA-DQB1	DQB1*05:01:01G	0
		tig00009488	HLA-DRB1	DRB1*10:01:01G	0
	TrioCanu HiFi	tig00004595|arrow|arrow	HLA-A	A*01:01:01G	0
		tig00004595|arrow|arrow	HLA-B	B*35:08:01G	0
		tig00004595|arrow|arrow	HLA-C	C*04:01:01G	0
		tig00004595|arrow|arrow	HLA-DQA1	DQA1*01:01:01G	0
		tig00004595|arrow|arrow	HLA-DQB1	DQB1*05:01:01G	0
		tig00004595|arrow|arrow	HLA-DRB1	DRB1*10:01:01G	0
		tig00004691|arrow|arrow	HLA-A	A*26:01:01G	0
		tig00004691|arrow|arrow	HLA-B	B*38:01:01G	0
		tig00004691|arrow|arrow	HLA-C	C*12:03:01G	0
		tig00004302|arrow|arrow	HLA-DQA1	DQA1*03:01:01G	0
		tig00004302|arrow|arrow	HLA-DQB1	DQB1*03:02:01G	0
		tig00004302|arrow|arrow	HLA-DRB1	DRB1*04:02:01	0
	Hi-C HiFi	HG002-S16-H1-000005F	HLA-A	A*01:01:01G	0

		HG002-S16-H1-000005F	HLA-B	B*35:08:01G	0
		HG002-S16-H1-000005F	HLA-C	C*04:01:01G	0
		HG002-S16-H1-000003F	HLA-DQA1	DQA1*01:01:01G	0
		HG002-S16-H1-000003F	HLA-DQB1	DQB1*05:01:01G	0
		HG002-S16-H1-000003F	HLA-DRB1	DRB1*03:96<br>DRB1*13:178	46
		HG002-S16-H2-000005F	HLA-A	A*26:01:01G	0
		HG002-S16-H2-000005F	HLA-B	B*38:01:01G	0
		HG002-S16-H2-000005F	HLA-C	C*12:03:01G	0
		HG002-S16-H2-000002F	HLA-DQA1	DQA1*03:01:01G	0
		HG002-S16-H2-000002F	HLA-DQB1	DQB1*03:02:01G	0
		HG002-S16-H2-000002F	HLA-DRB1	DRB1*04:02:01<br>DRB1*04:02:05	1
	Peregrine	000029F	HLA-A	A*01:01:01G	0
		000029F	HLA-B	B*38:01:01G	0
		000029F	HLA-C	C*12:03:01G	0
		000029F	HLA-DQA1	DQA1*03:01:01G	0
		000029F	HLA-DQB1	DQB1*03:02:01G	0
		000029F	HLA-DRB1	DRB1*04:02:01	0

Results for Peregrine, Canu and HiCanu assemblies (this paper), as well as results of a previous HiFi TrioCanu assembly (Wenger et al. 2019) and recent Hi-C (Garg et al. 2019) and StrandSeq (Porubsky et al. 2019) assemblies. The trio binning results are accurate and in phase across the entire MHC, capturing the locus in two contigs (one per haplotype). HiCanu assemblies include all expected alleles (without errors), but the reconstruction is more fragmented with a haplotype switch in the primary contig set between class I and class II genes in HG002 and after HLA-A in HG00733. The Hi-C based assembly from Garg et al. is in phase but has two non-0 edit distance genes, one of which matches neither haplotype. The StrandSeq phased assembly from Porubsky et al. only captures one version of the HLA-A and HLA-B genes, losing the alleles from other haplotype.

Supplementary Table 12. CHM13 centromeres identified by RepeatMasker

Assembly	Tig ID	Chromosome	Length (Mbp)	Centromere Start (Mbp)	Centromere End (Mbp)
CHM13 HiCanu 10kb	tig00001081	Chr11	81.40	35.37	38.68
CHM13 Canu 20kb	tig00002932	Chr16	5.02	1.30	3.15
	tig00000680	Chr11	81.34	35.37	38.62
	tig00001542	Chr19	51.89	24.75	28.54
CHM13 HiCanu 20kb	tig00006620	Chr2	49.17	2.3	4.9
	tig00000514	Chr3	199.46	90.0	96.6
	tig00000794	Chr7	147.14	44.5	51.2
	tig00018071	Chr8	129.87	31.2	34.7
	tig00001550	Chr10	112.08	38.9	43.1
	tig00001118	Chr12	126.71	34.0	38.1
	tig00018105	Chr16	26.11	4.0	7.0
	tig00006497	Chr19	46.04	21.7	28.1
	tig00005051	Chr20	65.33	25.3	31.3
HG002 HiCanu	tig00013421	Chr1	6.64	1.52	5.56
	tig00027658	Chr9	5.81	2.68	5.10
	tig00006752	Chr11	42.10	3.07	6.79
	tig00004350	Chr12	3.57	0.54	1.69
	tig00017535	Chr19	37.68	28.27	31.70
	tig00028481	ChrX	6.86	1.00	3.80
HG0733 HiCanu	tig00004390	Chr1	69.86	63.43	67.47
	tig00020838	Chr9	12.97	3.16	5.07
	tig00007347	Chr11	58.29	50.33	56.62

Positions of internal centromeres (at least 500 kbp away from the contig start/end) identified by RepeatMasker and manually adjusted based on visual inspection of RepeatMasker results. Peregrine did not assemble any internal centromeres on CHM13 so we excluded it from the table. Canu also performed worse than HiCanu and likely collapsed regions of the centromere (note they are all shorter than the same chromosome centromere in HiCanu) and thus we have excluded it from other genomes as well. Validation of diploid centromeres remains a challenge and is beyond the scope of this manuscript. The assemblies from HiCanu will mix variants from both the maternal and paternal haplotypes.
